# EPHA2/CD44-directed trafficking enhances endosomal leakiness and antisense therapy delivery

**DOI:** 10.1083/jcb.202507217

**Published:** 2026-08-11

**Authors:** Sergi Marco, Peter J. Walsh, Alexey S. Revenko, Tobias Schmidt, Peter A. Thomason, Lynn McGarry, A. Robert MacLeod, Sonam Ansel, Dina Tataran, Martin Bushell, Chiara Braconi, Jim C. Norman

**Affiliations:** 1 Cancer Research UK Scotland Institute, Glasgow, UK; 2 School of Cancer Sciences, University of Glasgow, Glasgow, UK; 3 Ionis Pharmaceuticals Inc., Carlsbad, CA, USA; 4 ADARx Pharmaceuticals Inc., San Diego, CA, USA; 5 Beatson West of Scotland Cancer Centre, Glasgow, UK

## Abstract

The potential for using therapeutic antisense oligonucleotides (ASOs) has been hampered by a lack of understanding of how they enter cells and subsequently access their targets. Endocytosis contributes to ASO uptake, but the machinery mediating subsequent ASO trafficking to permit suppression of their target mRNAs has not been described. Here, we show that direct ASO engagement with a scavenger receptor (CD44) activates the ERK-RSK axis to promote serine phosphorylation of a receptor tyrosine kinase (EPHA2). Serine phosphorylation of EPHA2 permits endocytosis, trafficking, and accumulation of ASOs in nuclear-captured endosomes. These endosomes are then subject to lipid peroxidation and become leaky, allowing ASOs to escape and effectively suppress target mRNA expression. Inhibition of stress granule-mediated repair of these leaky endosomes further enhances ASO effectiveness. These data identify an endocytic route to the nucleus which may be exploited to maximize the effectiveness of ASO-mediated therapies.

## Teaser

A scavenger receptor (CD44) and a receptor tyrosine kinase (EPHA2) join forces to mediate internalization of therapeutic ASOs into endosomes, which are then captured on the nuclear surface. Nuclear-captured endosomes become leaky, allowing ASO escape. This novel endocytic route provides insight into how ASO-mediated therapies may be optimized.

## Introduction

Antisense oligonucleotides (ASOs) are short single-stranded DNA molecules designed to target complementary mRNA and regulate its expression. Binding of an ASO to mRNA forms RNA–DNA hybrids that are recognized and degraded by the endonuclease RNase H1 (Cerritelli and Crouch, 2009; Nowotny et al., 2007), leading to reduced synthesis of the encoded protein. RNase H1 localizes to the nucleus and mitochondrion, where nascent RNAs are synthesized (Liang et al., 2017), but can also act in the cytoplasm where most mature transcripts reside. Thus, access to the appropriate cellular compartment is critical for ASO activity. To facilitate delivery, formulations such as liposomal encapsulation or sugar conjugation (Roberts et al., 2020) have been used; however, these approaches can cause unwanted side effects, including aberrant immune responses (Coelho et al., 2013; Moschos et al., 2007).

A newer generation of constrained ethyl-ASOs (cET-ASOs) enables vehicle-free delivery (Byrnes et al., 2023; Stein et al., 2010), thought to occur via endocytosis. Following uptake, cET-ASOs are sorted within the endosomal network, and their ability to escape from endosomal lumens into the cytoplasm or nucleus determines their capacity to engage target mRNAs and recruit RNase H1 (Linnane et al., 2019). Two major limitations are recycling to the extracellular space and trafficking to degradative compartments such as lysosomes, both of which reduce opportunities for endosomal escape (Kapustin et al., 2021; Linnane et al., 2019). Despite the importance of endocytic trafficking for ASO efficacy, the mechanisms governing their uptake and intracellular routing remain poorly understood.

We recently described a mechanism in which endosomes are directed to the nuclear vicinity and associate with nuclear pores (Marco et al., 2021). This “nuclear-capture” process is mediated by the cytoplasmic tail of the receptor tyrosine kinase EPHA2 (often overexpressed in tumors [Wykosky and Debinski, 2008]), through its interaction with the nuclear import machinery. Nuclear capture is mediated by phosphorylation of EPHA2 at Ser^897^, followed by endocytosis and positioning of EPHA2-containing endosomes near the nucleus.

High EPHA2 expression is often seen in tumor types which are driven by KRAS-activating mutations (Dunne et al., 2016; Markosyan et al., 2019; Mudali et al., 2006; Thaker et al., 2004). In pancreatic cancer, KRAS mutation is a key driver of tumor initiation, and mutant *Kras* deletion induces regression in preclinical models (Collins et al., 2012; Ying et al., 2012). We therefore investigated whether an EPHA2-dependent endocytic route leading to nuclear capture could be exploited to enhance uptake of a KRAS-targeting cET-ASO (cET-ASO^*Kras*^) in cells from pancreatic cancers.

## Results and discussion

### EPHA2 is required for productive uptake of *KRAS*-targeting ASOs

**Figure 1.**
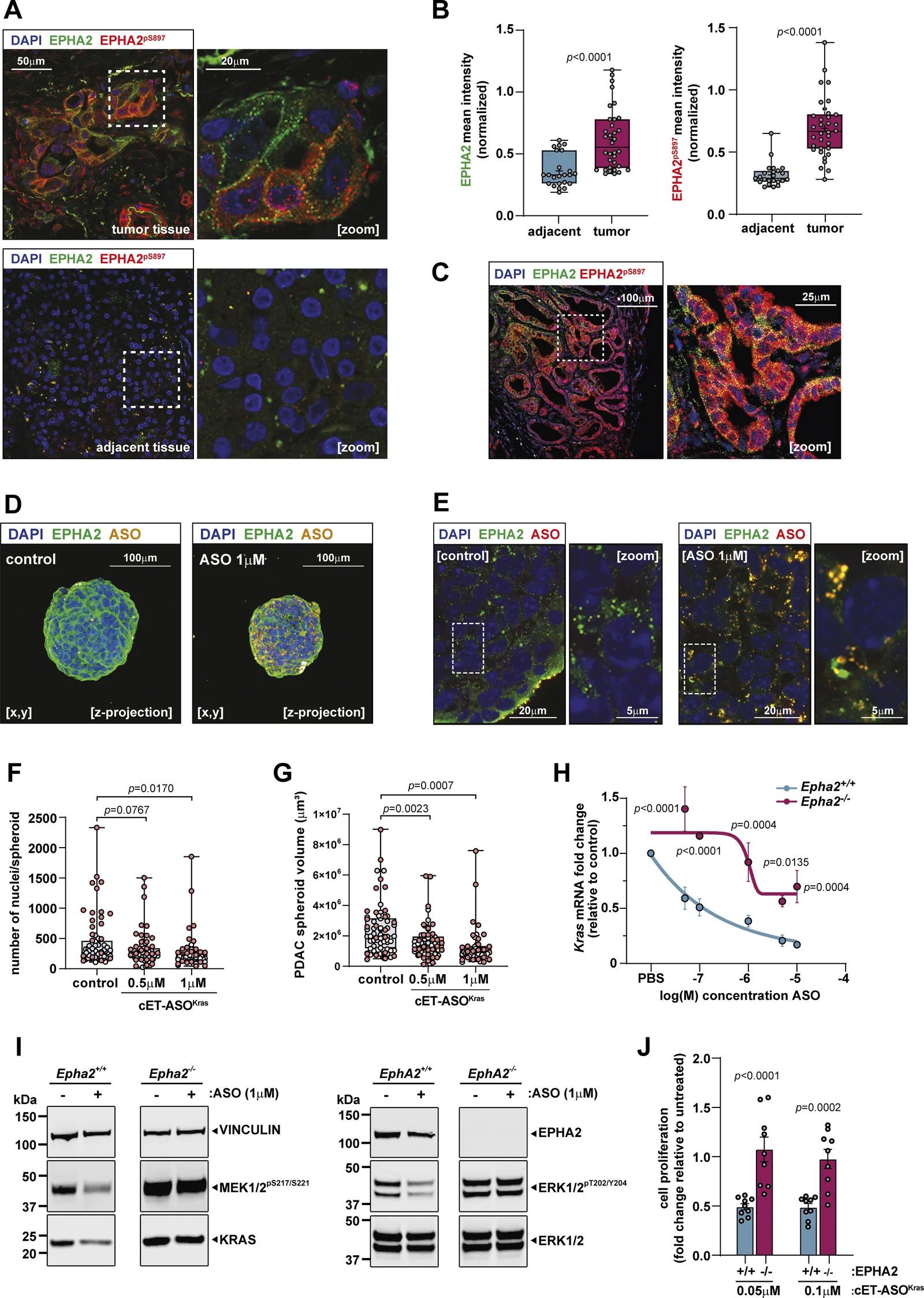
**EPHA2 is required for cET-ASO–mediated suppression of KRAS in PDAC. (A and B)** EPHA2 (green) and Ser^897^ phosphorylated EPHA2 (red) in PDAC patient tumor (top and right), and non-transformed adjacent tissue (bottom and left), and (B) their quantification in patient samples. Tumor *n* = 6; normal *n* = 8, unpaired *t* test. **(C)** EPHA2 (green) and Ser^897^ phosphorylated EPHA2 (red) in mouse PDAC (KPC). **(D)** EPHA2 (green) in KPC spheroids treated with cET-ASO^Kras^ (orange). **(E)** High-resolution micrograph of KPC cells either untreated (control) or treated with cET-ASO^Kras^ for 72 h. EPHA2 (green) and cET-ASO^Kras^ (red) staining are shown overlapping in intracellular vesicles. Side panels correspond to an image amplification of the area inside the white dotted line frames in the adjacent panels. **(F and G)** High-content image analysis of spheroid volume (G) and number of nuclei per spheroid (F) after 72-h treatment with *Kras*-targeting cET-ASO (ASO; 1 μM) of vehicle control (control). *n* = 3 independent experiments, one-way ANOVA (OWA), Dunnett. **(H)***Kras* mRNA expression in WT (*Epha2*^*+/+*^*,* blue) or *Epha2* knockout (*Epha2*^*−/−*^, magenta) KPC cells after 72-h treatment with cET-ASO^Kras^. Data are mean ± SEM, *n* = 5 independent experiments. **(I)** MEK and ERK activation status in KPC tumor-derived cell lines from either *Epha2*^*+/+*^ or *Epha2*^*−/−*^ mice after 72 h cET-ASO^Kras^ treatment. **(J)** KPC *Epha2*^*+/+*^ (blue) or *Epha2*^*−/−*^ (magenta) cell proliferation after treatment with *Kras*-targeting ASO for 96 h. Proliferation index expressed as the fold change of cell number relative to untreated cells. Data are mean ± SEM, *n* = 9 independent experiments. **(H and J)** Two-way ANOVA (TWA), Sidak. Source data are available for this figure: SourceData F1.

**Figure S1.**
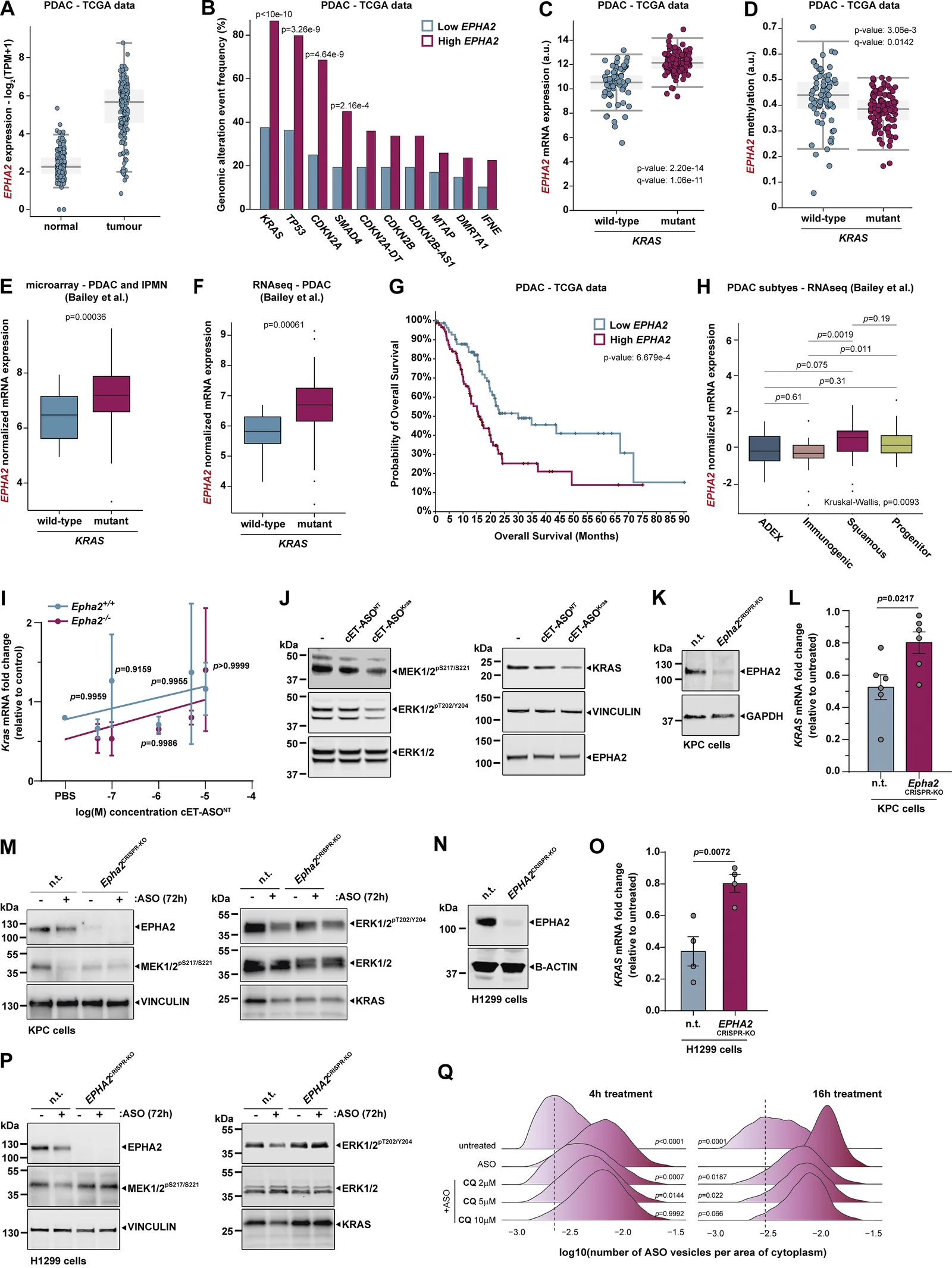
**Supplementary data related to Figs. 1 and 2. (A)** *EPHA2* expression in normal pancreas (TCGA and GTEx, *n* = 171) compared with expression in PDAC (TCGA, *n* = 179). **(B)** Analysis of the mutational frequency in the most common oncodrivers in PDAC (TCGA), comparing low *EPHA2* expression tumors (blue, *n* = 88) with high *EPHA2* expression tumors (magenta, *n* = 89). Two-sided Fisher exact test. **(C)** Comparison of the abundance of *EPHA2* mRNA in either *KRAS* WT (blue, *n* = 67) or *KRAS* mutant (magenta, *n* = 117) PDAC tumors (TCGA). Student’s *t* test. **(D)** Comparison of the promoter methylation levels on the *EPHA2* gene in either *KRAS* WT (blue, *n* = 67) or *KRAS* mutant (magenta, *n* = 117) PDAC tumors (TCGA). Student’s *t* test. **(E)** Comparison of the abundance of *EPHA2* mRNA in either *KRAS* WT (blue) or *KRAS* mutant (magenta) PDAC tumors (microarray data, PDAC, and intraductal papillary mucinous neoplasm—IPNM). *Kras* WT *n* = 24, *Kras* mutant *n* = 207. Student’s *t* test. **(F)** Comparison of the abundance of *EPHA2* mRNA in either *KRAS* WT (blue) or *KRAS* mutant (magenta) PDAC tumors (RNA-seq data^30^). *Kras* WT *n* = 11, *Kras* mutant *n* = 84. Student’s *t* test. **(G)** Survival plot; probability of overall survival of PDAC patients comparing patients with low *EPHA2* expression (blue, *n* = 88) and those with high *EPHA2* expression (magenta, *n* = 89). Log-rank test. **(H)***EPHA2* mRNA expression comparison across the different PDAC subtypes as defined by Bailey et al. (2016). ADEX *n* = 16, immunogenic *n* = 25, progenitor *n* = 30, and squamous *n* = 25. Kruskal–Wallis. **(I)***Kras* mRNA expression after 72 h of treatment with the indicated concentrations of cET-ASO^NT^ in WT (*Epha2*^*+/+*^*,* blue line) or *Epha2* knockout (*Epha2*^*−/−*^, magenta line) KPC cells. Two-way ANOVA, Sidak multiple comparison test, *n* = 3 individual experiments. **(J)** Western blots comparing the effect of cET-ASO^NT^ and cET-ASO^Kras^ on MEK1/2 and ERK1/2 phosphorylation and KRAS expression in KPC cells. Vinculin was used as a loading control. **(K)** Western blotting of KPC cell lysates obtained from either KPC cells transduced with a nontargeting sgRNA (n.t.) or an *Epha2*-targeting sgRNA (Epha2^CRISPR-KO^), showing the depletion of EPHA2 expression in the latter. GAPDH was used as a loading control. **(L)***Kras* mRNA expression in either nontargeting or 60 Epha2^CRISPR-KO^ KPC cells after treatment with 0.5 μM cET-ASO^Kras^ for 72 h. Data are mean ± SEM, *n* = 6 independent experiments, Student’s *t* test. **(M)** Western blotting of either nontargeting or Epha2^CRISPR-KO^ KPC cells after treatment with 0.5 μM cET-ASO^Kras^ for 72 h, showing the effect of the ASO on MEK1/2 and ERK1/2 phosphorylation and KRAS expression. Vinculin was used as a loading control. **(N)** Western blotting of H1299 (non-small cell lung carcinoma) cell lysates obtained from either H1299 cells transduced with either a nontargeting sgRNA (n.t.) or an *EPHA2*-targeting sgRNA (EPHA2^CRISPR-KO^), showing the depletion of EPHA2 expression in the latter. β-actin was used as a loading control. **(O)***KRAS* mRNA expression in either nontargeting or EPHA2^CRISPR-KO^ H1299 cells after treatment with 0.5 μM cET-ASO^Kras^ for 72 h. Data are mean ± SEM, *n* = 4 independent experiments, Student’s *t* test. **(P)** Western blotting of either nontargeting or EPHA2^CRISPR-KO^ H1299 cells after treatment with 0.5 μM cET-ASO^Kras^ for 72 h, showing the effect of the ASO on MEK1/2 and ERK1/2 phosphorylation and KRAS expression. Vinculin was used as a loading control. **(Q)** Density plots of the number of ASO-positive vesicles (number per area of cytoplasm) in cells treated either with vehicle or the indicated concentrations of chloroquine in combination with either vehicle or ASO (5 μM) for either 4 or 16 h. Data are expressed as log10-transformed, *n* = 3 independent experiments; all conditions are compared with vehicle-ASO–treated cells, ANOVA, and Tukey post hoc test. Source data are available for this figure: SourceData FS1.

In human pancreatic ductal adenocarcinoma (PDAC), EPHA2 is strongly expressed in tumor nodules but largely absent in adjacent normal tissue (Fig. 1, A and B; and Fig. S1 A). Key PDAC drivers—mutated *KRAS*, *TP53*, *CDKN2A*, and *SMAD4*—correlate with increased EPHA2 (Fig. S1, B–F), and EPHA2 levels associate with reduced overall survival and the aggressive squamous subtype (Fig. S1, G and H). Notably, human PDACs exhibit EPHA2 Ser^897^ phosphorylation (Fig. 1, A and B), a requirement for nuclear capture of endosomes (Marco et al., 2021). Similarly, tumors from the KPC mouse (*Kras*^*G12D/+*^; *LSL-Trp53*^*R172H/+*^; *Pdx1-Cre*), which mimics metastatic squamous PDAC (Gopinathan et al., 2015), express abundant phospho-Ser^897^ EPHA2 (Fig. 1 C). To test KRAS dependency for PDAC growth, KPC-derived spheroids were treated with the *Kras*-targeting cET-ASO, cET-ASO^*Kras*^. cET-ASO^*Kras*^ was taken up by KPC cells, and this significantly reduced spheroid volume and cell number (Fig. 1, D–G), confirming that ASO-mediated *Kras* inhibition can oppose PDAC growth in 3D culture.

To investigate EPHA2’s role in cET-ASO^*Kras*^ uptake into PDAC, we used tumor cell lines from KPC mice that were either WT (KPC-*Epha2*^*+/+*^*)* or knockout (KPC-*Epha2*^*−/−*^*) for Epha2*. In KPC-*Epha2*^*+/+*^ cells, cET-ASO^*Kras*^ reduced *Kras* mRNA dose dependently, reaching 50% inhibition at ∼0.1 μM (Fig. 1 H). Contrastingly, in KPC-*Epha2*^*−/−*^ cells, cET-ASO^*Kras*^’s ability to suppress *Kras* expression was reduced by >100-fold (Fig. 1 H). Western blotting confirmed that 1 μM cET-ASO^*Kras*^ suppressed KRAS protein levels and downstream MEK/ERK phosphorylation in KPC-*Epha2*^*+/+*^, but not KPC-*Epha2*^*−/−*^ cells (Fig. 1 I). Accordingly, cET-ASO^*Kras*^ inhibited growth only in KPC-*Epha2*^*+/+*^ cells (Fig. 1 J). Importantly, a nontargeting ASO (cET-ASO^*NT*^) affected neither *Kras* expression nor MEK/ERK signalling (Fig. S1, I and J). EPHA2’s necessity for ASO-mediated *Kras* targeting was further validated by CRISPR-mediated *Epha2* deletion in KPC (Fig. S1, K–M) and human H1299 lung carcinoma cells (Fig. S1, N–P).

### EPHA2-mediated nuclear capture is required for cET-ASO^*Kras*^ to suppress *KRAS* expression

**Figure 2.**
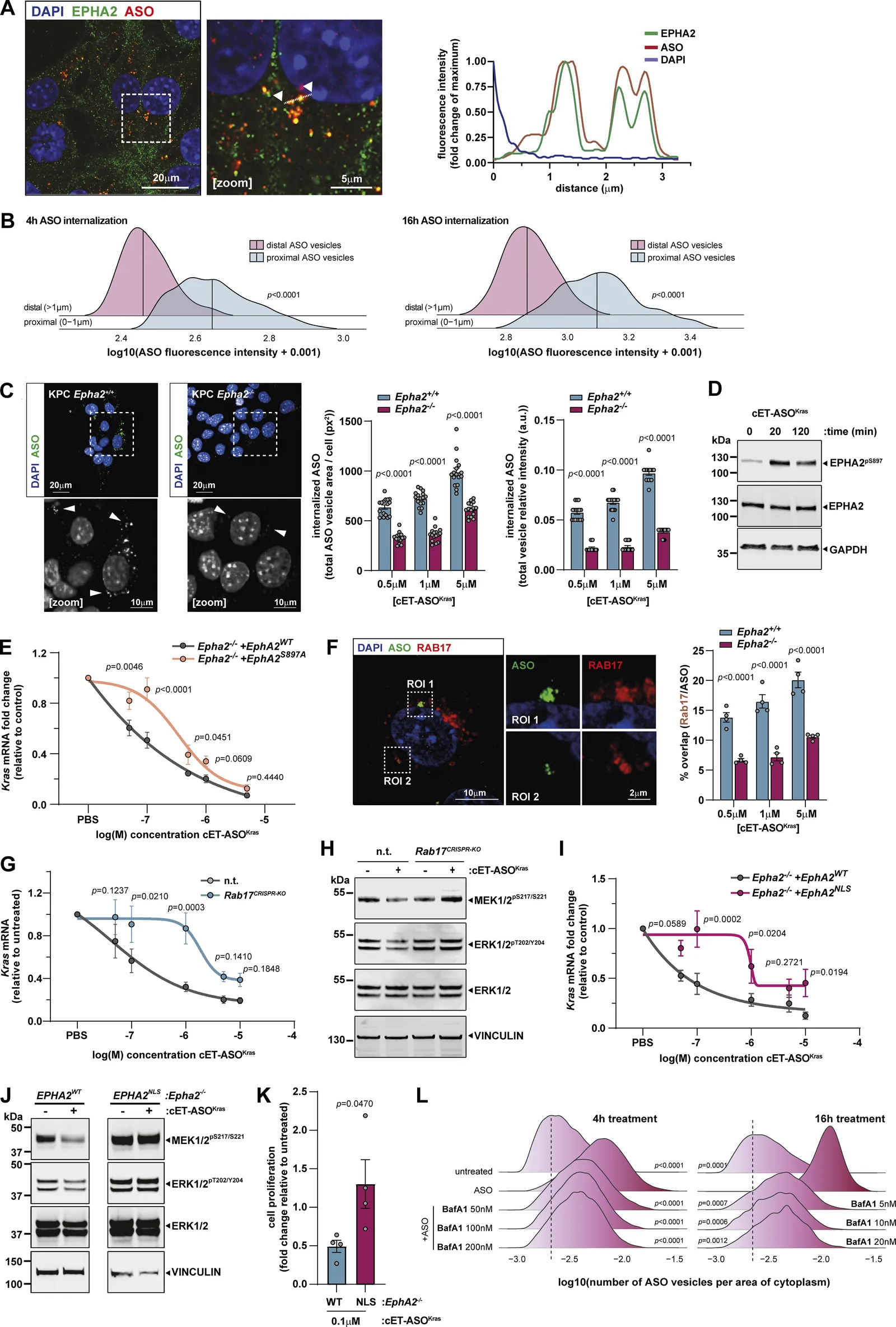
**EPHA2-mediated trafficking and nuclear capture dictates cET-ASO** ^**Kras**^ **efficacy. (A)** Internalization for 16 h of cET-ASO^Kras^ (red) in KPC cells showing overlap of cET-ASO^Kras^ and EPHA2 (green) in internalized vesicles (white arrows). **(B)** Quantification and binning of cET-ASO^Kras-positive^ vesicle-nucleus distance into proximal (0–1 µm) or distal (>1 µm) categories. Density plots show ASO fluorescence intensities per ASO-positive vesicle expressed as log-transformed pseudo-counts. *n* = 3 independent experiments. Paired *t* test. **(C)** cET-ASO^Kras-positive ^intracellular vesicle area per cell and relative intensity per vesicle in either KPC *Epha2*^*+/+*^ (blue bars) or *Epha2*^*−/−*^ (magenta bars) cells after ASO treatment, *n* = 16 cells. **(D)** EPHA2 phospho-Ser^897^ in KPC cells with and without treatment with 1 μM cET-ASO^Kras^. **(E)***Kras* mRNA expression after 72 h of cET-ASO^Kras^ treatment in EPHA2 WT rescue (*Epha2*^*−/−*^*+ EPHA2*^*WT*^, gray) or EPHA2^S897A^ mutant rescue (*Epha2*^*−/−*^*+ EPHA2*^*S897*^, salmon) KPC cells; data are mean ± SEM, *n* = 4 independent experiments. **(F)** Overlap between cET-ASO^Kras^ (green) and RAB17-positive vesicles (red) in KPC cells; data are mean ± SEM, *n* = 4 independent experiments. **(G)***Kras* mRNA expression in *Epha2*^*+/+*^ nontargeting-CRISPR (gray line) and *Rab17*-CRISPR^KO^ KPC cells (blue line) after 72 h of cET-ASO^Kras^ treatment; data are mean ± SEM, *n* = 6 independent experiments. **(H)** MEK1/2 and ERK1/2 induction in *Epha2*^*+/+*^ nontargeting or *Rab17*-CRISPR^KO^ KPC cells after 72 h of cET-ASO^Kras^ treatment. Vinculin is a loading control. **(I)***Kras* mRNA expression after 72 h of treatment with cET-ASO^Kras^ in EPHA2 WT rescue (*Epha2*^*−/−*^*+ EPHA2*^*WT*^, gray) or EPHA2 NLS mutant rescue (*Epha2*^*−/−*^*+ EPHA2*^*NLS*^, magenta) KPC cells; data are mean ± SEM, *n* = 7 independent experiments. **(J)** MEK1/2 and ERK1/2 activation in *Epha2*^*−/−*^*+ EPHA2*^*WT*^ and *Epha2*^*−/−*^*+ EPHA2*^*NLS*^ KPC cells after 72 h of cET-ASO^Kras^. Vinculin is a loading control. **(K)** Cell proliferation after cET-ASO^Kras^ (96 h) treatment in either *Epha2*^*−/−*^*+ EPHA2*^*WT*^ (blue bar) or *Epha2*^*−/−*^*+ EPHA2*^*NLS*^ (magenta bar) KPC cells; data are mean ± SEM, *n* = 4 independent experiments. TWA, Sidak for all panels. **(L)** Density plots of ASO-positive vesicles (vesicle number per area of cytoplasm) in cells treated either with vehicle or the indicated concentrations of bafilomycinA1 in combination with either vehicle or ASO (5 μM) for either 4 or 16 h. Data are expressed as log10-transformed, *n* = 3 independent experiments. All conditions are compared with vehicle-ASO–treated cells, ANOVA, and Tukey post hoc test. Source data are available for this figure: SourceData F2. TWA, two-way ANOVA.

We hypothesized that EPHA2 facilitates cET-ASO^*Kras*^ endocytosis and subsequent nuclear capture of ASO-containing endosomes to suppress *Kras* expression. Indeed, endocytosed cET-ASO^*Kras*^ localized to nuclear-proximal, EPHA2-positive vesicles 4 h after addition (Fig. 2, A and B), and ASO internalization was significantly reduced in *Epha2* knockout cells (Fig. 2 C), confirming EPHA2’s role in ASO endocytosis and trafficking.

We previously linked EPHA2-mediated nuclear capture to EphA2-Ser^897^ phosphorylation, Rab17-dependent trafficking, and NLS-driven nuclear capture. Investigating the requirement for these processes for productive ASO uptake, we report the following:

1 EphA2 phosphorylation: cET-ASO^*Kras*^ increased EphA2-Ser^897^ phosphorylation (Fig. 2 D), and a phospho-defective EPHA2 mutant (EPHA2^S897A^) failed to fully rescue ASO-driven *Kras* suppression in *Epha2*^*−/−*^ cells (Fig. 2 E).
2 Rab17-dependent trafficking: EPHA2 knockout reduced ASO trafficking to RAB17-positive endosomes (Fig. 2 F). Consistently, cET-ASO^*Kras*^*’s* ability to suppress *Kras* and its downstream signalling was compromised in RAB17 knockout cells (Fig. 2, G and H).
3 Nuclear capture: While EPHA2^WT^ restored cET-ASO^*Kras*^ activity in *Epha2*^*−/−*^ cells, an NLS mutant (EPHA2^NLS^)—which prevents nuclear capture (Marco et al., 2021)—did not (Fig. 2, I and J). Accordingly, cET-ASO^*Kras*^ suppressed growth in EPHA2^WT^ but not EPHA2^NLS^ cells (Fig. 2 K).

Finally, bafilomycinA1 or chloroquine reduced endosomal accumulation of ASO (Fig. 2 L and Fig. S1 Q), indicating endosomal acidification is required for trafficking to nuclear-proximal vesicles. Together, these data demonstrate that cET-ASO^*Kras*^ efficacy requires EPHA2-dependent endocytosis and Rab17-dependent trafficking to nuclear-captured endosomes.

### Scavenger receptors trigger EPHA2-dependent uptake and trafficking of ASOs

**Figure 3.**
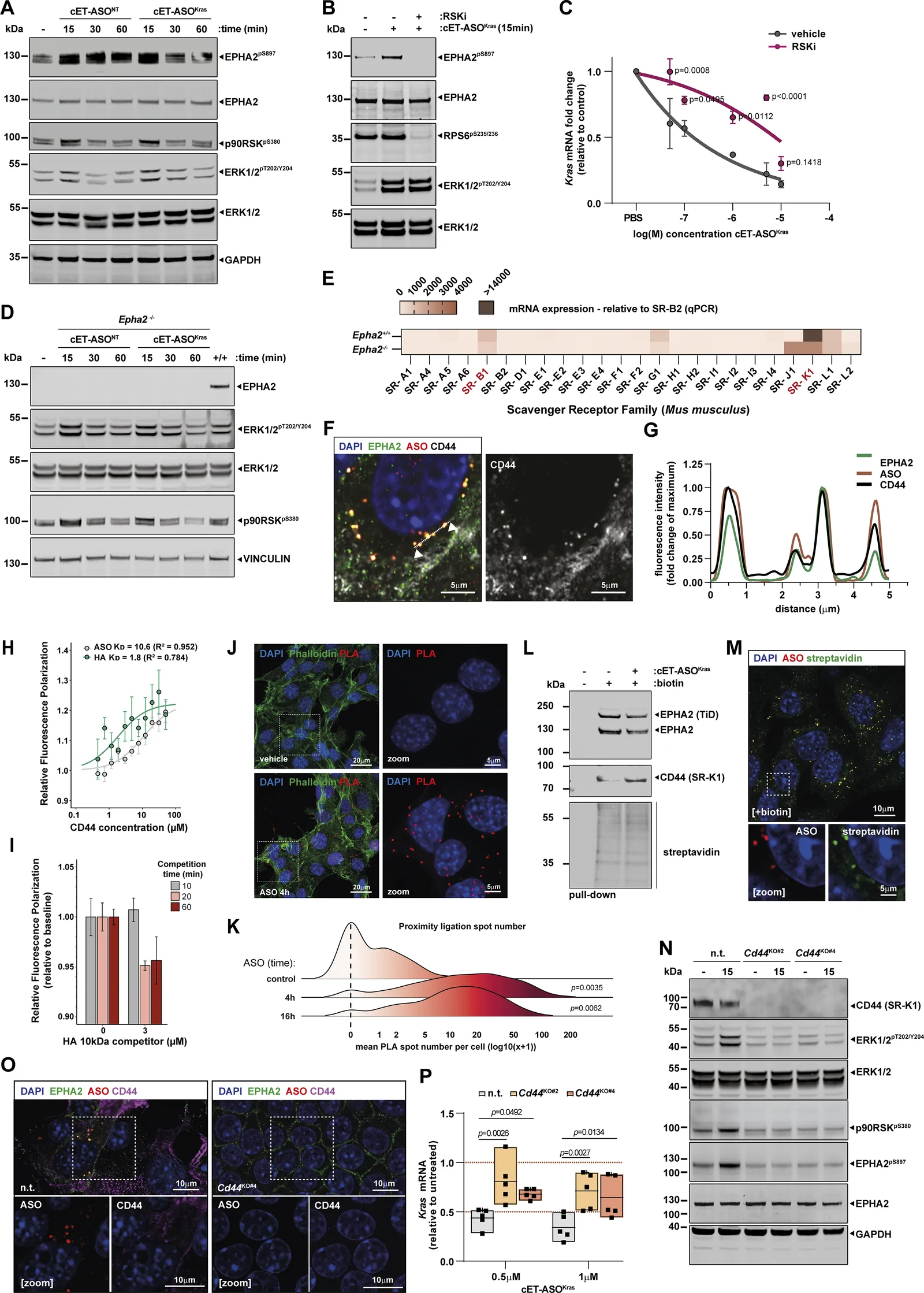
**SRs trigger EPHA2-dependent uptake and trafficking of ASOs. (A)** Nontargeting cET-ASO (cET-ASO^NT^) or cET-ASO^Kras^ induction of EPHA2 Ser^897^, p90RSK, and ERK1/2 phosphorylation in KPC cells. GAPDH is a loading control. **(B)** Effect of the RSK inhibitor LHJ685 (RSKi) on the cET-ASO^Kras^ -induced phosphorylation of EPHA2 Ser^897^ in KPC cells. **(C)***Kras* mRNA expression 72-h following treatment with the indicated concentrations of cET-ASO^Kras^ (72 h) in vehicle (gray line) or RSK inhibitor-treated (10 μM, magenta line) KPC cells; data are mean ± SEM, *n* = 3 independent experiments. **(D)** cET-ASO^NT^ or cET-ASO^Kras^ induction of p90RSK and ERK1/2 phosphorylation in *Epha2*^*−/−*^ KPC cells. Western blotting was performed on the same samples that were used for the experiment presented in supplemental Fig. S2 C. Thus, the vinculin blot used as a loading control in Fig. 3 D is also used as a sample control for supplemental Fig. S2 C. **(E)** Heatmap showing the levels of mRNA expression of the SR family in either *Epha2*^*+/+*^ (top) or *Epha2*^*−/−*^ (bottom) KPC cells. **(F and G)** Internalization of cET-ASO^Kras^ (16 h, red), EPHA2 (green), and CD44 (white) in KPC cells. **(H)** Fluorescence polarization of either cET-ASO-Cy3 or HA-Cy3 (10 kDa) in the presence of the indicated concentrations of recombinant-hCD44. Receptor–ligand affinity data are fitted using a 1:1 binding model. Fitting (R^2^) and dissociation values (K_D_) are shown for each molecule; data are mean ± SEM, *n* = 3 independent experiments. **(I)** Displacement of cET-ASO-Cy3 from the cET-ASO-Cy3/rhCD44 complex by increasing concentrations of unlabelled HA (10 kDa) is shown; data are mean ± SEM, *n* = 3 independent experiments. **(J and K)** Fluorescence micrographs of Duolink proximity ligation (PLA, red) between cET-ASO and CD44 in KPC cells (K), and quantification of the average number of PLA spots per cell after cET-ASO addition for 4 or 16 h (K). Data are expressed as log10(x+1) spots per cell, *n* = 3 independent experiments, ANOVA Tukey post hoc test. **(L)** Pulldown of biotin-labelled CD44 from EPHA2-TurboID expressing cells after cET-ASO^Kras^ treatment for 16 h. **(M)** cET-ASO^Kras^^-positive^ internalized vesicles (red) and biotin-labelled targets (green) in EPHA2-TiD expressing KPC cells. **(N)** cET-ASO^Kras^–induced activation of ERK1/2, p90RSK and EPHA2 phospho-Ser^897^ in *Cd44*-knockout KPC cells. GAPDH is a loading control. **(O)** Overlap of intracellular CD44 and internalized ASO in control and *Cd44*^*KO*^ KPC cells. **(P)***Kras* mRNA expression in either nontargeting or *Cd44*^*CRISPR-KO*^ KPC cell lines treated with the indicated concentrations of cET-ASO^Kras^ (72 h), *n* = 5 independent experiments, TWA Sidak for all panels. Source data are available for this figure: SourceData F3. TWA, two-way ANOVA.

**Figure S2.**
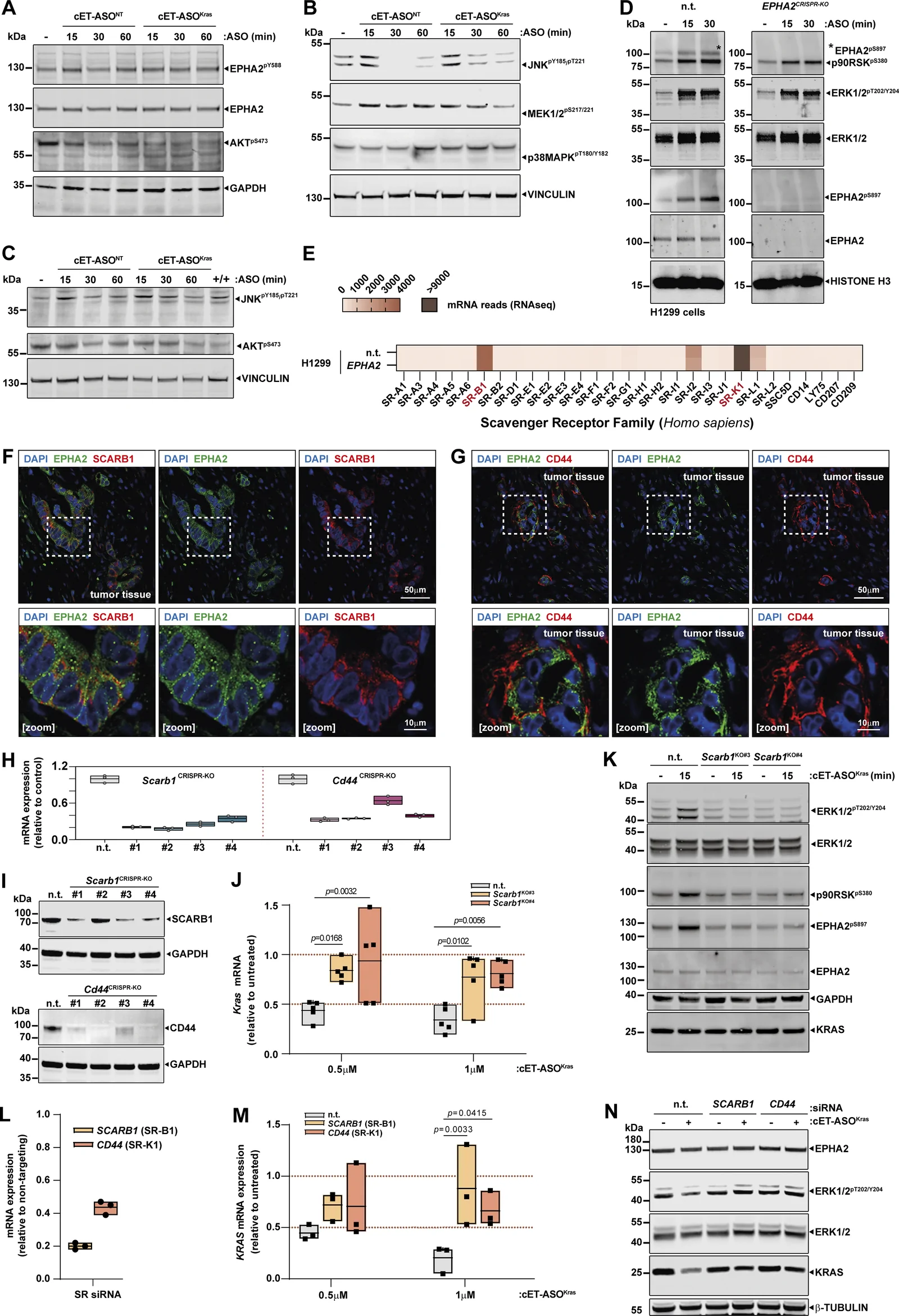
**Supplementary data related to Fig. 3. (A)** Western blotting of either nontargeting cET-ASO^NT^ or cET-ASO^Kras^–treated KPC cells, showing the absence of phosphorylation induction on EPHA2 Tyr^588^ and AKT Ser^473^. GAPDH was used as a loading control. **(B)** Western blotting of either nontargeting cET-ASO^NT^ or cET-ASO^Kras^–treated KPC cells, showing the induction of phosphorylation on JNK Tyr^185^ and Thr^221^, MEK1/2 Ser^217^ and Ser^221^, as well as the lack of induction of phosphorylation on p38MAPK Thr^180^ and Tyr^182^. Vinculin was used as a loading control. **(C)** Western blotting in either cET-ASO^NT^ or cET-ASO^Kras^–treated KPC cells, showing the induction of phosphorylation on JNK Tyr^185^ and Thr^221^ and the lack of induction of phosphorylation on AKT Ser^473^ in KPC *Epha2*^−/−^ cells. Western blotting was performed on the same samples that were used for the experiment presented in Fig. 3 D. Thus, the vinculin blot used as a loading control in Fig. 3 D is also used as a sample control in this panel. **(D)** Western blotting showing the induction of the phosphorylation of EPHA2 Ser^897^, p90RSK Ser^380^, and ERK1/2 Tyr^202/204^ in cET-ASO^Kras^–treated *EPHA2*^+/+^ and *EPHA2*^CRISPR-KO^ H1299 cells. * Band corresponds to the band previously obtained by blotting for EPHA2 phospho-Ser^897^. Histone H3 was used as a loading control. **(E)** Heatmap showing levels of mRNA expression (RNA-seq normalized reads) of each member of the SR family in either *EPHA2*^*+/+*^ (top) or *EPHA2-*siRNA depleted (bottom) H1299 cells. Red text corresponds to the highest expressed genes both in KPC and H1299 cells. Outlier values are indicated in dark brown. **(F)** PDAC patient tumor tissue immunostaining. Top panels show the staining of EPHA2 (green) and SCARB1 (red) within the tumor nodules. Bottom panels correspond to amplification of the area contained in the white dotted line frame. **(G)** PDAC patient tumor tissue immunostaining. Top panels show the staining of EPHA2 (green) and SR-K1 (CD44, red) within the tumor nodules. Bottom panels correspond to amplification of the area contained in the white dotted line frame. **(H)***Scarb1* and *Cd44* mRNA expression levels in KPC cells transduced with either a nontargeting sgRNA (n.t.) or four different sgRNAs targeting either *Scarb1* (*Scarb1*^CRISPR-KO^) or *Cd44* (*Cd44*^CRISPR-KO^). Dots represent *n* = 3 independent experiments. **(I)** Western blotting showing the protein expression of either SCARB1 in nontargeting and *Scarb1*^CRISPR-KO^ KPC cells, or CD44 in *Cd44*^CRISPR-KO^ KPC cells. GAPDH is a loading control. **(J)***Kras* mRNA expression in either nontargeting or *Scarb1*^*CRISPR-KO*^ KPC cell lines treated with either 0.5 μM or 1 μM cET-ASO^Kras^ for 72 h. Two-way ANOVA, Dunnett multiple comparison test, *n* = 5 independent experiments. **(K)** Western blotting showing the effect of *Scarb1* depletion in *Scarb1*^CRISPR-KO^ KPC cell lines on the cET-ASO^Kras^–induced activation of ERK1/2 Tyr^202/204^, p90RSK Ser^380^, and EPHA2 Ser^897^ phosphorylation. GAPDH was used as a loading control. **(L)***SCARB1* and *CD44* mRNA expression in H1299 cells transduced with either a nontargeting siRNA or a pooled siRNA for each respective scavenging receptor. Results expressed as the fold change of each mRNA in nontargeting transduced cells, *n* = 3 independent experiments. **(M)***KRAS* mRNA expression in H1299 cells transduced with either a nontargeting siRNA or a pooled siRNA for each respective scavenging receptor, treated with either 0.5 μM or 1 µM cET-ASO^Kras^ for 72 h. Two-way ANOVA, Tukey multiple comparison test, *n* = 3 independent experiments. **(N)** Western blotting showing the effect of depletion of *SCARB1* and *CD44* SRs in H1299 cell lines on the MAPK signalling activation and *KRAS* expression after 72 h of cET-ASO^Kras^ addition. β-tubulin was used as a loading control. Source data are available for this figure: SourceData FS2.

Since cET-ASO increases EPHA2 Ser^897^ phosphorylation (Fig. 2 D), and phospho-defective EPHA2^S897A^ does not support cET-ASO^*Kras*^ uptake (Fig. 2 E), we investigated the signalling underlying ASO-driven EPHA2 phosphorylation. Various kinases, like AKT and p90RSK, phosphorylate Ser^897^ following growth factor or stress cues (Hamaoka et al., 2018; Harada et al., 2015; Miao et al., 2015; Zhou et al., 2015). Western blotting showed KPC cells rapidly activate p90RSK, but not AKT, in response to cET-ASO (Fig. 3 A and Fig. S2 A). MAPK signalling (MEK, ERK, and JNK, but not p38MAPK), which typically activates p90RSK, also increased (Fig. 3 A and Fig. S2 B). Consistently, a p90RSK inhibitor LHJ685 (RSKi) blocked cET-ASO–driven p90RSK activation (evidenced by reduced pSer^235/236^-RPS6) and EPHA2 Ser^897^ phosphorylation (Fig. 3 B), and p90RSK inhibition decreased cET-ASO^*Kras*^’s ability to suppress *Kras* levels (Fig. 3 C). Alongside this, we found that cET-ASO still activated ERK, JNK, and p90RSK in EPHA2 knockout KPC or H1299 cells (Fig. 3 D; and Fig. S2, C and D). Taken together, these data indicate that cET-ASO–driven activation of MAPK and p90RSK signalling is upstream of EPHA2-Ser^897^ phosphorylation.

We next investigated if a receptor-mediated mechanism drives cET-ASO–induced MAPK/p90RSK signalling and EPHA2 phosphorylation. Scavenger receptors (SRs) mediate myeloid antigen uptake and hepatocyte lipid clearance (Terpstra et al., 2000; Yu et al., 2015). SRs also internalize polyanionic molecules like ASOs (Miller et al., 2016; Tanowitz et al., 2017) and initiate MAPK signalling (Grewal et al., 2003; Yang et al., 2020a). Our screen of KPC (Fig. 3 E) and H1299 (Fig. S2 E) cells identified SCARB1 (SR-B1) and CD44 (SR-K1) as highly expressed in both cell types. CD44 and SCARB1 localized at or near the plasma membrane in human PDAC tumors (Fig. S2, F and G); notably, CD44 expression is highest in the most aggressive squamous PDAC subtype (Bailey et al., 2016).

As cET-ASO^*Kras*^ was endocytosed into perinuclear endosomes positive for EPHA2 and CD44 (Fig. 3, F and G), we considered whether CD44 and cET-ASOs directly associate to form a receptor–ligand complex. Fluorescence polarization spectroscopy showed that Cy3-labelled cET-ASO and the canonical CD44 ligand, hyaluronic acid (HA), both directly bind purified CD44 with micromolar affinity (of 10.8 and 1.8 µM, respectively) (Fig. 3 H), and unlabelled HA displaced Cy3-cET-ASO from CD44 (Fig. 3 I), suggesting they compete for the same site. Consistently, proximity ligation assays showed that cET-ASO^*Kras*^ and CD44 associate *in situ* (Fig. 3, J and K). To test if cET-ASOs can physically connect EPHA2 with CD44, we used an EPHA2-TurboID fusion to perform proximity-dependent biotinylation. cET-ASO^*Kras*^ addition increased CD44 biotinylation by EPHA2-TurboID (Fig. 3 L) and the appearance of biotin-labelled proteins in intracellular vesicles (Fig. 3 M). Together, these data indicate that cET-ASOs interact directly with SRs to promote physical association and co-endocytosis of an EPHA2–SR complex.

We next investigated how SRs influence cET-ASO internalization, MAPK/p90RSK signalling, and KRAS suppression. CRISPR-mediated deletion of *Cd44* or *Scarb1* in KPC cells (Fig. S2, H and I) blocked cET-ASO–driven activation of MAPKs and p90RSK, as well as EPHA2 Ser^897^ phosphorylation (Fig. 3 N and Fig. S2 K). Accordingly, vesicles containing ASOs and/or internalized EPHA2 were undetectable in *Cd44*-deficient cells (Fig. 3 O). Furthermore, deleting *Cd44* or *Scarb1* prevented cET-ASO^*Kras*^ from suppressing *Kras* expression in KPC (Fig. 3 P and Fig. S2 J) and H1299 cells (Fig. S2, L–N). These data indicate that cET-ASOs activate SRs to trigger p90RSK signalling, CD44-EPHA2 association, and EPHA2 Ser^897^ phosphorylation. This process then facilitates the co-trafficking of cET-ASO, SRs, and phospho-EPHA2 to juxta-nuclear endosomes, enabling productive ASO uptake and targeting *Kras* mRNAs.

### EPHA2 is required for cET-ASO trafficking to leaky endosomes

**Figure 4.**
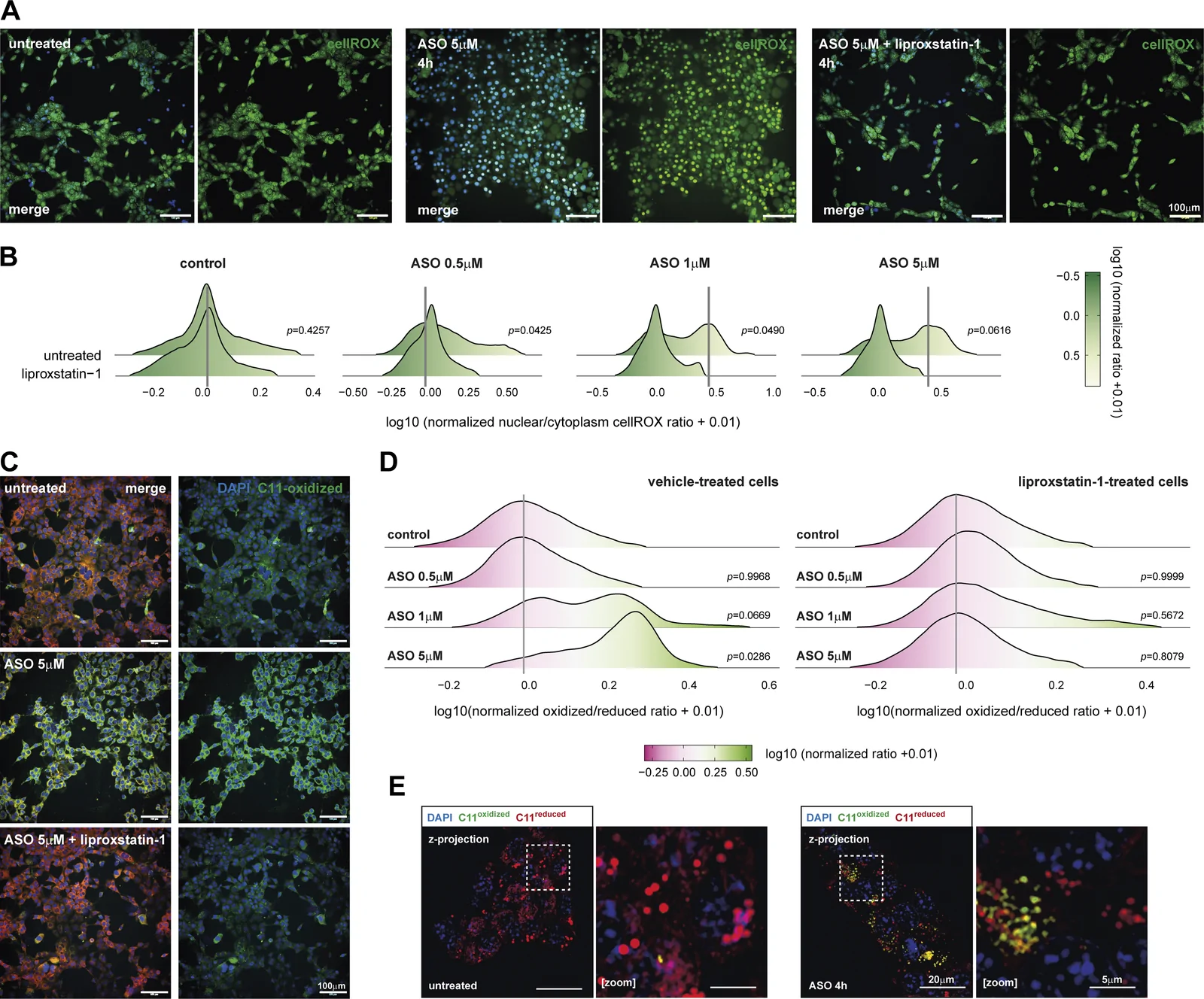
**EPHA2 is required for trafficking of cET-ASOs to leaky endosomes. (A)** Fluorescence micrographs of KPC cells treated either with vehicle (untreated) or cET-ASO^Kras^ (5 μM) in combination with vehicle or liproxstatin-1 (1 μM) for 4 h. Nuclei (blue) and CellROX green staining are displayed (left panels are merged, right panels are CellROX only). **(B)** Quantification of CellROX green nuclear translocation. Density plot data are expressed as nuclear/cytoplasmic ratio (log10-transformed pseudo-counts), *n* = 5 independent experiments, ANOVA Tukey post hoc test. **(C)** Fluorescence micrographs of KPC cells treated either with vehicle or cET-ASO^Kras^ (5 μM) in combination with vehicle (untreated) or liproxstatin-1 (1 μM) for 4 h. Nuclei (blue) and C11-bodipy (red and green denoting reduced and oxidized lipids respectively) staining (left panels are merged, right panels are oxidized C11-bodipy only) are displayed. **(D)** Quantification of C11-bodipy oxidation. Density plot data are expressed as the oxidized/nonoxidized ratio (log10-transformed pseudo-counts), *n* = 4 independent experiments, ANOVA Tukey post hoc test. **(E)** Airyscan micrographs of KPC cells treated with either vehicle or cET-ASO^Kras^ (5 μM) and stained with C11-bodipy.

Endosomal escape of ASOs to the cytoplasm, through mechanisms which remain largely unclear, is required for mRNA targeting. Reactive oxygen species (ROS) can damage internal membranes to increase their permeability and allow endosomal escape—a process which is thought to be linked to antigen cross-presentation (Bhardwaj et al., 2023; Canton et al., 2021; Dingjan et al., 2016). Reports that CD44 signalling is involved in both antigen presentation and ROS production in endo-lysosomal compartments (Niemietz and Brown, 2023; Vachon et al., 2006) further prompted us to investigate if ASO uptake evokes endomembrane damage. Treating KPC cells with cET-ASO^*Kras*^ significantly increased ROS levels, as measured by nuclear translocation of CellROX green (Fig. 4, A and B). To detect lipid peroxidation (LP)—a key driver of ROS-mediated membrane damage—we used the C11-Bodipy probe (Drummen et al., 2002). cET-ASO^*Kras*^ addition increased LP (Fig. 4, C and D) specifically within intracellular vesicles (Fig. 4 E). Moreover, the radical-trapping antioxidant liproxstatin-1 blocked these ASO-induced increases in both ROS and LP (Fig. 4, A and D).

**Figure 5.**
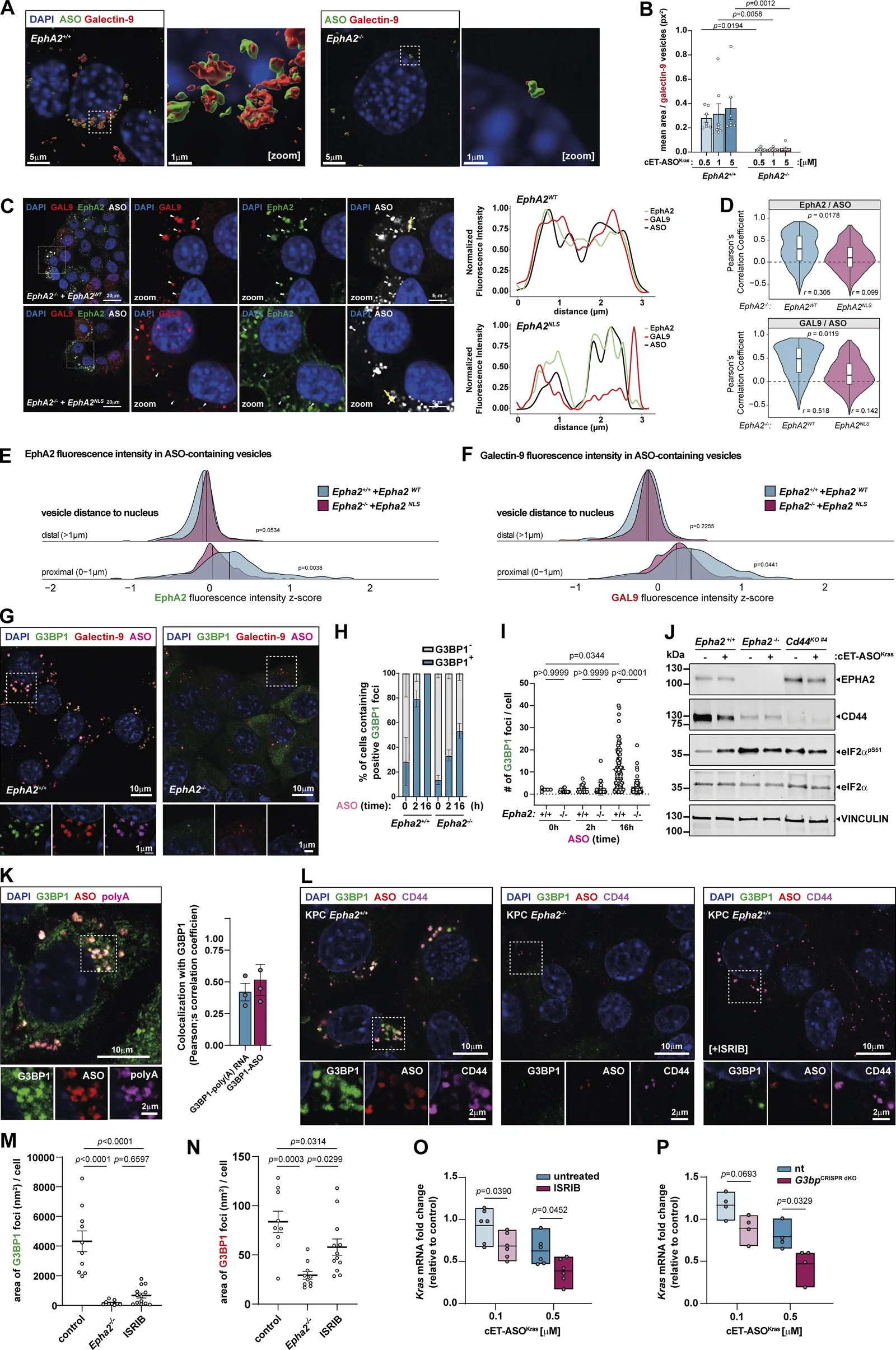
**EPHA2 is required for trafficking of cET-ASOs to leaky endosomes. (A and B)** Galectin-9 (red) overlaps with cET-ASO^Kras^ (green) in vesicles, and (B) quantification in either *Epha2*^*+/+*^ (blue bars) or *Epha2*^*−/−*^ (magenta bars) KPC cells, after 16-h treatment with the indicated concentrations of cET-ASO^Kras^ (16 h); data are mean ± SEM, *n* = 7 fields. TWA, Tukey. **(C)** Fluorescence micrographs (Airyscan) of *Epha2*^*−/−*^ KPC cells expressing either *Epha2*^*WT*^ or *Epha2*^*NLS*^ (green) in combination with galectin-9 (red) following addition of cET-ASO^Kras^ (16 h, white). Arrowheads show instances of ASO accumulation. Fluorescence profiles (right panels) corresponding to the yellow lines in the micrographs. **(D)** Quantification of the colocalization (Pearson’s correlation coefficient) between EPHA2 and ASOs or galectin-9 and ASO in intracellular vesicles corresponding to the groups in C, *n* = 3 independent experiments, unpaired *t* test. **(E and F)** Quantification and binning of cET-ASO^Kras^ vesicle-nucleus distance into proximal (0–1 µm) or distal (>1 µm) categories. Density plots show EPHA2 (E) and galectin-9 (F) normalized fluorescence intensity z-scores per vesicle, *n* = 3 independent experiments, ANOVA, Tukey post hoc test. **(G)** 3D-reconstruction of galectin-9 (red) overlap with cET-ASO^Kras^ (magenta) and G3BP1-GFP (green) in *Epha2*^*+/+*^ or *Epha2*^*−/−*^ KPC cells. (H) Percentage of cells that contain at least one G3BP1-positive condensate in either *Epha2*^*+/+*^ or *Epha2*^*−/−*^ KPC cells after treatment with cET-ASO^Kras^. **(I)** Average G3BP1-positive foci per cell in either *Epha2*^*+/+*^ or *Epha2*^*−/−*^ KPC cells after cET-ASO^Kras^ treatment, *n* = 3 independent experiments, OWA, Tukey. **(J)** Effect on phosphoSer^51^ eIF2α following cET-ASO^Kras^ (4 h) addition in either *Epha2*^*+/+*^ or *Epha2*^*−/−*^ KPC cells. Vinculin is a loading control. **(K)** Distribution of poly-d(T) (determined by *in situ* hybridization; magenta) and G3BP1-GFP (green) after 16-h treatment with cET-ASO^Kras^ (red) in KPC cells. **(L)** Overlap between G3BP1-GFP, cET-ASO^Kras^, and CD44 in either *Epha2*^*+/+*^, *Epha2*^*−/−*^, or ISRIB (1 μM)-treated *Epha2*^*+/+*^ KPC cells, *n* = 3 independent experiments. **(M and N)** Quantification of the average sum of the area of intracellular G3BP1-GFP condensates (K) and ASO vesicles (L) per cell, in cET-ASO^Kras^–positive vesicles, *n* = 3 independent experiments, Tukey. **(O and P)** Effect of ISRIB and *G3bp1/G3bp2* CRISPR knockout (G3BP^dKO^) on cET-ASO^Kras^*–*induced reduction of *Kras* mRNA expression in KPC cells, *n* = 6 and 4 independent experiments, respectively, TWA, Sidak. Source data are available for this figure: SourceData F5. OWA, one-way ANOVA; TWA, two-way ANOVA.

**Figure S3.**
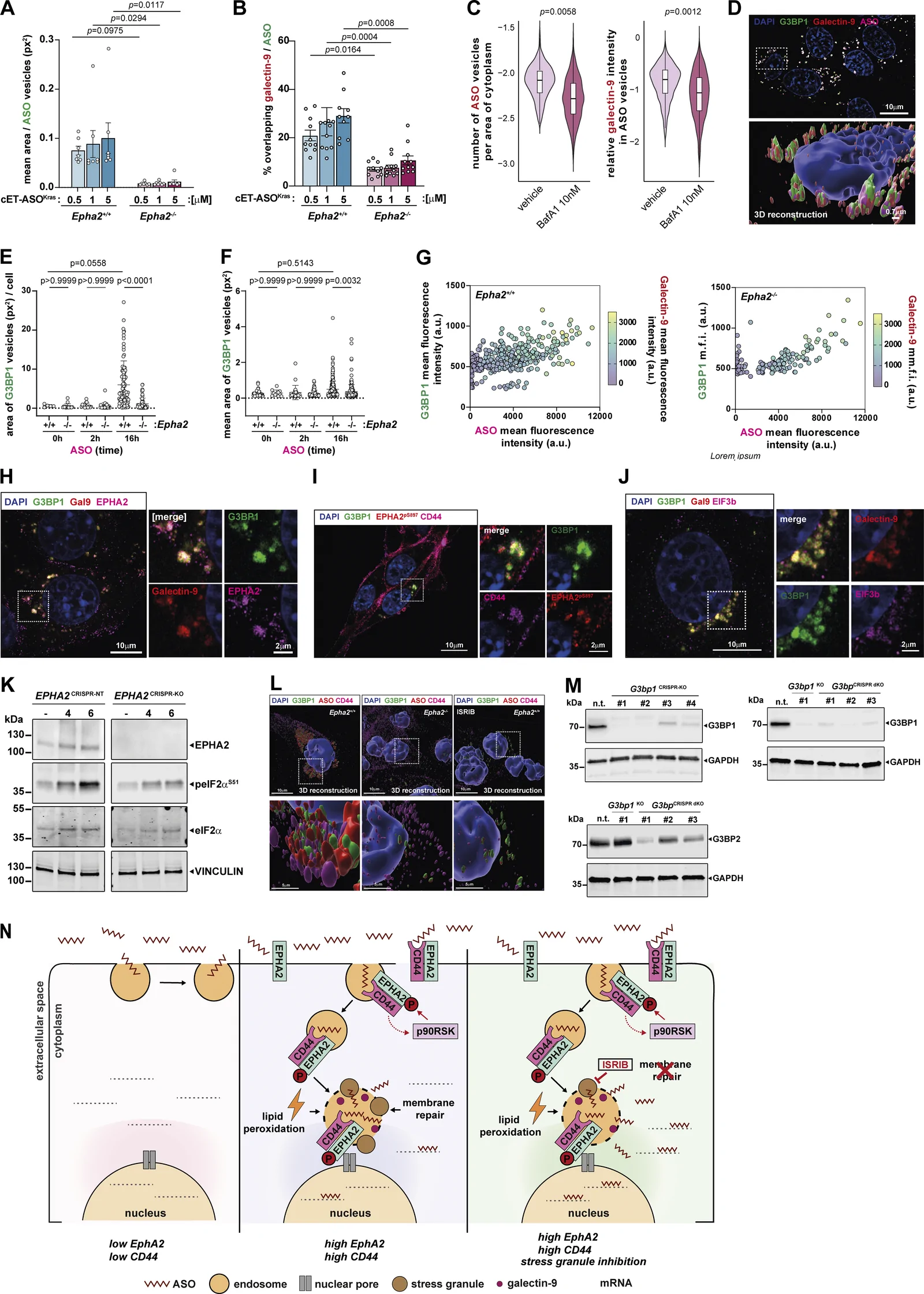
**Supplementary data related to Fig. 5.** **(A)** Quantification of the mean area of ASO-positive vesicles in either *Epha2*^*+/+*^ (blue bars) or *Epha2*^*−/−*^ (magenta bars) KPC cells, after treatment with either 0.5, 1, or 5 μM of cET-ASO^Kras^ for 16 h. One-way ANOVA, Tukey multiple comparison test, *n* = 7. **(B)** Quantification of the mean area size of galectin-9–positive vesicles in either *Epha2*^*+/+*^ (blue bars) or *Epha2*^*−/−*^ (magenta bars) KPC cells, after treatment with either 0.5, 1, or 5 μM of cET-ASO^Kras^ for 16 h. One-way ANOVA, Tukey multiple comparison test, *n* = 11. **(C)** Quantification of the number of ASO vesicles per area of cytoplasm (left panel) and galectin-9 fluorescence intensity (right panel), in cells treated with either vehicle or bafilomycinA1 (10 nM) for 4 h in combination with cET-ASO^Kras^ (5 μM). *n* = 3 independent experiments, ANOVA, Tukey post hoc test. **(D)** 3D-reconstruction of galectin-9 (red) overlap with cET-ASO^Kras^ (magenta) and G3BP1-GFP (green) in *Epha2*^*+/+*^ or *Epha2*^*−/−*^ KPC cells. **(E and F)** Average sum of the area of G3BP1-positive structures per cell (E) and mean area of individual G3BP1 vesicles (F) in either *Epha2*^*+/+*^ or *Epha2*^*−/−*^ KPC cells after incubation with cET-ASO^Kras^ for 0, 2, or 16 h. Dots correspond to different fields of view obtained from three individual experiments. One-way ANOVA—Tukey test. **(G)** Scatter plot of the mean intensity values for G3BP1, ASO, and galectin-9 fluorescence for each cell in either *Epha2*^*+/+*^ (left panel) or *Epha2*^*−/−*^ (right panel) KPC cells after incubation with cET-ASO^Kras^ for 16 h. **(H)** Micrographs of immunofluorescence confocal imaging showing the overlap between G3BP1-GFP, EPHA2, and galectin-9 in intracellular structures in KPC cells treated with cET- ASO^Kras^ for 16 h. **(I)** Micrographs of confocal immunofluorescence imaging showing the overlap between G3BP1-GFP, internalized EPHA2^pS897^, and intracellular CD44 in KPC cells treated with cET-ASO^Kras^ for 16 h. Image zooms correspond to the area inside the corresponding dotted line boxes. **(J)** Micrographs of confocal immunofluorescence imaging showing the overlap between G3BP1-GFP, galectin-9, and eIF3b fluorescence in intracellular structures in KPC cells treated with cET-ASO^Kras^ for 16 h. **(K)** Western blotting of either nontargeting (EPHA2^CRISPR-NT^) or EPHA2^CRISPR-KO^ H1299 cells showing the effect on eIF2α phosphorylation after incubation with ASO cET-ASO^Kras^ for 4 or 6 h. Vinculin was used as a loading control. **(L)** 3D reconstruction of immunofluorescence confocal imaging showing the overlap between G3BP1-GFP (green), internalized cET-ASO^Kras^ (red), and intracellular CD44 (pink) in either *Epha2*^*+/+*^, *Epha2*^*−/−*^, or ISRIB (1 μM)-treated *Epha2*^*+/+*^ KPC cells. **(M)** Western blotting showing the protein expression of G3BP1 in nontargeting (n.t.) and KPC cells transduced with four different sgRNA-targeting *G3bp1* in KPC cells (*G3bp1*^CRISPR-KO^, upper left panel); western blotting showing the protein expression of G3BP2 in nontargeting (n.t.), *G3bp1*^CRISPR-KO#1^ and *G3bp1*^CRISPR-KO#1^ KPC cells transduced with three different sgRNAs targeting *G3bp2* (*G3bp1/2*^CRISPR-KO^, upper right panel); western blotting showing the protein expression of G3BP1 in nontargeting (n.t.), *G3bp1*^CRISPR-KO#1^, and *G3bp1*^CRISPR-KO#1^ KPC cells transduced with three different sgRNAs targeting *G3bp2* (*G3bp1/2*^CRISPR-KO^, lower left panel). GAPDH was used as a loading control. **(N)** Diagram depicting the endocytic pathway driven by CD44 and EphA2 transporting ASOs from the extracellular space into the perinuclear region and enabling their release from nuclear-captured leaky endosomes. The left panel depicts the situation when both EPHA2 and CD44 levels are low, and there is little internalization of ASO. The center panel summarizes events when CD44 and EPHA2 levels are both high. ASO directly engages with CD44, which then drives activation of p90RSK to phosphorylate EPHA2 on Ser^897^. This promotes internalization of ASO into EPHA2/CD44 positive vesicles, which are then trafficked to the juxta-nuclear region in a Rab17-dependent manner. ASO-containing endosomes are then captured on the nuclear surface via an interaction between the cytotail of EPHA2 and the nuclear pore complex. ASO-driven increase in ROS production and the resulting LP leads to endosomal membrane damage. Subsequently, these vesicles become leaky, allowing escape of the ASO, despite the activation of repair mechanisms under the ISR, such as SG formation. When SG assembly is inhibited using ISRIB, this repair process is compromised, and nuclear-captured endosomes become even more leaky, as denoted in the right panel. Source data are available for this figure: SourceData FS3.

While endosomal leakiness promotes ASO release, it also recruits repair effectors like ESCRT components or galectins (galectin-3, -8, and -9) (Gros et al., 2022; Hedlund et al., 2023; Jia et al., 2018; Jia et al., 2020; Skowyra et al., 2018; Wittrup et al., 2015). We detected galectin-9 as a proxy for leaky membranes and thus identified potential sites of cET-ASO endosomal escape. In KPC-*Epha2*^*+/+*^, but not KPC-*Epha2*^*−/−*^, cells, cET-ASOs increased the size and overlap of galectin-9/ASO-positive vesicles (Fig. 5, A and B; Fig. S3, A and B). BafilomycinA1 treatment reduced galectin-9 recruitment to ASO-positive vesicles (Fig. S3 C), suggesting endosomal maturation is required for ASO-induced leakiness. Finally, cells expressing nuclear capture–defective EPHA2 (*EphA2*^*NLS*^) showed decreased colocalization of cET-ASO with EPHA2 and galectin-9 (Fig. 5, C and D). This reduction was most pronounced in nuclear-proximal vesicles (Fig. 5, E and F), indicating EPHA2 nuclear capture is essential for trafficking ASOs to leak-prone endosomes.

LP and endo-lysosomal damage drive liquid–liquid phase separation, triggering assembly of ribonucleoprotein and stress granules (SGs) (Balakrishnan and Kenworthy, 2024; Yang et al., 2020b). Moreover, G3BP1, a core SG component, has recently been shown to be recruited to leaky endosomes to facilitate their repair (Bussi et al., 2023). Following cET-ASO addition, many nuclear-proximal structures were triply positive for cET-ASO, galectin-9, and G3BP1 (Fig. S3 D). This G3BP1 recruitment was EPHA2-dependent; KPC *Epha2*^*−/−*^ cells largely failed to form G3BP1 condensates (Fig. 5, G and H), which, if present, were reduced in size and number (Fig. 5 I and Fig. S3, E–G). Furthermore, EPHA2, EPHA2^pS897^, and CD44 overlapped with G3BP1/galectin-9 structures (Fig. S3, H and I), linking them to the nuclear-capture pathway. Collectively, these data indicate that cET-ASOs, via SR signalling, promote EPHA2^pS897^-dependent trafficking to nuclear-proximal compartments. These compartments subsequently become leaky, enabling endosomal escape and productive cET-ASO^*Kras*^ uptake.

### EPHA2-dependent uptake of cET-ASO^*Kras*^ is enhanced by inhibiting endosomal repair

Cellular stresses activate kinases like PKR, PERK, GCN2, and HRI, increasing eIF2α phosphorylation and triggering SG assembly as part of the integrated stress response (ISR) (Klein et al., 2022). Addition of cET-ASO increased phospho-eIF2α in KPC cells (Fig. 5 J), but not in *Epha2-*or *Cd44*-knockout KPC cells. This suggests EPHA2-dependent cET-ASO internalization and delivery to nuclear-captured endosomes drives SG assembly. Fluorescence *in situ* hybridization using a poly-d(T) probe showed close colocalization of polyadenylated mRNAs and G3BP1 with internalized cET-ASOs (Fig. 5 K). Similarly, cET-ASO addition induced G3BP1/galectin-9–positive structures associated with the SG marker EIF3b (Fig. S3 J) (Jayabalan et al., 2016). Thus, cET-ASO–positive endosomes in EPHA2-expressing cells associate closely with *bona fide* SG markers.

Beyond regulating mRNA translation, SGs recently were shown to plug endo-lysosomal membrane holes caused by mycobacteria, restricting their leakage and pathogenicity (Bussi et al., 2023; Jia et al., 2022). We proposed that SG formation at damage sites similarly limits cET-ASO efficacy. To test this, we used ISRIB (ISR inhibitor), which opposes SG assembly by blocking eIF2α phosphorylation effects (Rabouw et al., 2019; Sidrauski et al., 2015). As expected, ISRIB inhibited cET-ASO–driven SG assembly and G3BP1 recruitment without affecting endosomal ASO uptake (Fig. 5, L–N; and Fig. S3, K and L). Notably, ISRIB enhanced the ability of cET-ASO^*Kras*^ to suppress *Kras* mRNA expression (Fig. 5 O). SG assembly can be opposed by combined knockout of its core components G3BP1 and G3BP2 (Yang et al., 2020b). CRISPR-mediated deletion of both genes (*G3bp*^CRISPR dKO^) (Fig. S3 M) significantly enhanced cET-ASO^*Kras*^ efficacy in suppressing *Kras* expression in KPC cells (Fig. 5 P). These data indicate that EPHA2-dependent trafficking to leaky, nuclear-proximal endosomes is essential for efficient target gene suppression, and that ASO efficiency is enhanced by inhibiting SG-mediated endosomal repair mechanisms (Fig. S3 N).

The efficacy of genetic medicine can be limited by poor endosomal escape. We identified a pathway where cET-ASOs bind a SR, CD44, which cooperates with EPHA2 to drive ASO uptake and trafficking to nuclear-proximal endosomes. These nuclear-captured compartments become leaky, allowing ASO escape into the cytoplasm (Fig. S3 N). Pharmacological or genetic inhibition of SG-mediated membrane repair further enhances this efficacy. SR-dependent activation of p90RSK-driven EPHA2 phosphorylation initiates this process. While SRs traditionally mediate antigen uptake and endosomal leakage for MHC presentation in immune cells (Barth et al., 2008; Gros et al., 2022; Urban et al., 2001), in PDAC cells, SRs are more likely to support nutrient uptake and stemness (Guerrero-Rodríguez et al., 2022). Thus, we propose that a receptor which has been selected for its ability to support tumor stemness and growth may be exploited as a gatekeeper to an endocytic pathway capable of delivering therapeutic molecules to aggressive tumors. CD44 may promote endosomal leakiness by influencing iron uptake, either through Fe^3+^-loaded HA recruitment or TfR upregulation (Ando et al., 2025; Caneque et al., 2025). This Fe^3+^ can drive Fenton reaction-mediated LP, permeabilizing the ASO-loaded compartments (Saimoto et al., 2025). Thus, CD44 facilitates productive ASO uptake both by trafficking molecules to specific endosomes and by promoting the membrane damage necessary for their release.

While RTKs like EGFR are known to facilitate cET-ASO endocytosis (Wang et al., 2018), subsequent trafficking determines if ASOs can reach target mRNAs. Our evidence shows EPHA2 drives both cET-ASO uptake and trafficking to leaky endosomes. Crucially, *Kras*-mutant tumors, including most PDAC, express high levels of EPHA2 (Fig. S1 B), making them ideal candidates for cET-ASO therapy. Although EPHA2 normally functions during development by engaging ephrin ligands leading to autophosphorylation on tyrosine and trafficking to lysosomes, in cancer high levels of unengaged EPHA2 in cancer allow EPHA2-Ser^897^ phosphorylation in response to specific cues (Gundry et al., 2017; Marco et al., 2021). Rather than terminating signalling in lysosomes (Hiramoto-Yamaki et al., 2010), this phosphorylation enables nuclear capture of EPHA2-containing endosomes (Marco et al., 2021). The proximity of these leaky vesicles to the nucleus, governed by EPHA2’s NLS sequences, mechanistically links nuclear capture to endosomal leakiness.

Endosomal leakiness varies by compartment; early/recycling endosomes offer the highest probability for cargo escape (Paramasivam et al., 2022). Consequently, directing ASOs to these compartments can boost their activity (Finicle et al., 2023). Our data show cET-ASOs accumulate in RAB17, RAB11, RAB14, and RCP-positive recycling endosomes, linking the recycling characteristics of nuclear-captured vesicles with leakiness. Furthermore, blocking endosomal acidification/maturation—which prevents cargo delivery to recycling endosomes—compromised cET-ASO accumulation, confirming that this type of compartment facilitates cytosolic leakage.

We also identified SG recruitment to damaged endosomes occurring in response to ASO-induced leakiness. Traditionally known as mRNA translation regulators (Buchan and Parker, 2009; Moon et al., 2019), SGs are now implicated in endomembrane repair (Bussi et al., 2023; Jia et al., 2022). Our finding that chemical SG inhibition increases cET-ASO efficacy suggests that pharmacological tools targeting the translation machinery could be exploited to enhance the delivery of endocytosed therapeutics.

## Materials and methods

### Tissue immunofluorescence

Immunofluorescence staining was performed on paraffin-embedded tissue sections of patient pancreatic adenocarcinoma (PAAD; PDAC) tumors or KPC mouse PDAC tumor sections. Human tissues were retrieved through patients undergoing surgery with curative intent within Greater Glasgow and Clyde NHS hospitals through the Glasgow Biorepository (N.526) (*n* = 7). KPC tissue samples were obtained from archived paraffin blocks used in previous studies (Gundry et al., 2017).

Briefly, paraffin was removed from tumor sections containing slides using sequential steps with xylene, 100, 95, and 80% ethanol washes and a final step with H_2_O rehydration. Antigen retrieval was performed in citrate buffer, pH 6.0, and samples were placed in a water bath at 100°C for 20 min and allowed to cool down at RT for a further 20 min. Slides were washed three times with Tris-buffered saline (TBS), and samples were incubated with blocking buffer (1% normal goat serum and 0.1% Triton X-100 in TBS) for 1 h at RT. Primary antibodies were incubated ON at 4°C. The next day, slides were washed three times with TBS, and the corresponding secondary antibodies together with DAPI were incubated on each slide for 1 h at RT. Next, samples were washed three times with TBS buffer, and coverslips were mounted afterward using Fluoromount G mounting medium. Samples were visualized using a Zeiss LSM880 microscope with a 20×/0.8 NA objective and Zen Black 2.3 software; sequential excitation was used, and pixel size was 0.06 µm. Image analysis to measure the mean intensity of each fluorophore was performed across the different patient tissues in different fields of view for each patient using Fiji-ImageJ software (2.6.0).

### Cell culture

KPC and H1299 human non-small cell lung carcinoma cell lines were cultured in DMEM, high glucose, and GlutaMAX, supplemented with 10% FBS (vol/vol). Cells were cultured at 37°C in a controlled humidified environment containing 5% CO_2_, and cultures were split every 3–4 days using trypsin/EDTA solution. Cells were tested for the presence of mycoplasma, and H1299 cells were validated using the Promega GenePrint 10 system (STR multiplex assay) at the Molecular Technology Services (CRUK-Scotland Institute).

### KPC PDAC spheroids

Agarose 9 × 9 micro-molds were prepared as per the manufacturer’s instructions (Microtissues 3D Petri Dish micro-mold, Merck) and placed in each well of a 12-well cell culture plate. KPC cells were trypsinized, and 4.5 × 10^5^ cells were seeded into each agarose micro-mold using 190 ul of medium. Cells were allowed to enter the micro-wells for 2 h at 37°C, and the medium was topped up immediately after covering the whole micro-mold. Cells were allowed to form spheroids for 3 days, and on DIV3, cET-ASO^Kras^ was added to each corresponding well. On DIV7, the spheroids were fixed while inside the micro-molds using 4% paraformaldehyde for 1 h at RT. Next, the spheroids were popped out from the microwells using PBS, collected, and transferred to an empty 96-well plate for manipulation. Spheroids were then incubated with blocking buffer (0.1% Triton X-100, 2% BSA, and PBS) for 30 min at RT. Primary antibodies were diluted in blocking buffer and incubated with the spheroids ON at 4°C. The next day, three 30-min washes with blocking buffer were performed for each set of spheroids, and then samples were incubated with the corresponding secondary antibodies as well as DAPI. Samples were incubated ON at 4°C. The next day, the spheroids were washed three times for a 30-min period each time, and finally samples were mounted with Fungi solution (60% glycerol, 2.5 M fructose, and H_2_O) on a slide, using a frame seal (Bio-Rad) and a coverslip. Samples were visualized using either a Zeiss LSM880 confocal microscope as above or a Revvity Opera Phenix automated confocal microscope using a 20×/1.0NA water immersion objective (part HH14000421). Z-stacks using a 1 µm step were obtained for each spheroid and used to measure spheroid cell number and spheroid total volume. Data shown correspond to the individual spheroids pooled from independent experiments. Image analysis was performed using Harmony High-Content image analysis software (Revvity).

### cET-ASO dose–response curve

KPC or H1299 cells were seeded at a density of 1.5 × 10^5^ cells for each condition in a 6-cm plate. Cells were allowed to attach and proliferate overnight. The next day, cells were treated with either vehicle (PBS) or cET-ASOs at 0.05, 0.1, 1, 5, and 10 μM concentrations for 72 h. Next, RNA was harvested using Trizol (Thermo Fisher Scientific). 1 μg of RNA was used for RT-PCR to produce cDNA (High-capacity RNA to cDNA kit, Thermo Fisher Scientific) following the manufacturer’s recommended protocol. *Kras* mRNA expression was assessed using quantitative PCR (qPCR) using Quantinova SYBR Green RT-PCR Kit (Qiagen), a CFX1000 thermocycler (Bio-Rad), and CFX Maestro software (Bio-Rad). RPLP0 ribosomal RNA was used as a reference gene. *Kras* mRNA expression is expressed relative to untreated cells for each condition across independent experiments.

### Western blotting

KPC or H1299 cells were seeded at a density of 3 × 10^5^ cells in a 6-cm plate for long experiments (72 h) or 1 × 10^6^ cells for short experiments (15 min–16 h). Cells were treated with either vehicle (PBS) or cET-ASOs accordingly, and protein was harvested using RIPA buffer supplemented with protease and phosphatase inhibitors (Halt Protease and Phosphatase Inhibitor Cocktail, Thermo Fisher Scientific) at the endpoint. Protein lysates were sonicated, and insoluble fractions were discarded after centrifugation at 8,900 rcf at 4°C for 5 min. Protein concentration was measured using a BCA assay (Thermo Fisher Scientific). Uniformly concentrated samples were separated by electrophoresis using NuPAGE 4–12% Bis-Tris gels (Thermo Fisher Scientific), and proteins were transferred to a nitrocellulose membrane using the Trans-Turbo transfer system (Bio-Rad). Membranes were further incubated with blocking buffer (5% BSA in TBS 0.1% Tween20) for 1 h at RT. Corresponding antibodies were incubated ON at 4°C on a rocker. Membranes were washed the following day three times with TBS-T at RT. The corresponding secondary antibodies were incubated in blocking buffer for 1 h at RT. Three washes with TBS-T were subsequently performed, and protein expression was evaluated either using the Odyssey CLx Imager (Li-Cor) or using an ECL substrate (SuperSignal West Femto substrate, Thermo Fisher Scientific) and imaging with a ChemiDoc imaging system (Bio-Rad).

### Cell proliferation assay

KPC cells were seeded in 96-well optical bottom plates using 750 cells per well. Cells were allowed to attach and proliferate overnight. The next day, cells were treated with vehicle or the corresponding dose of cEt-ASO and allowed to proliferate for a further 72 h. At endpoint, cells were fixed with 4% paraformaldehyde solution in PBS, permeabilized using 0.1% Triton X-100 in PBS, and stained using DAPI. Plates were imaged using the Opera Phenix imaging system (Revvity) using a 5×/0.16NA objective (part HH14000402), and cell counting was performed using Harmony high-content image analysis software (Revvity). Data are expressed as proliferation index, corresponding to the fold change of cell growth between the control cells and the cET-ASO–treated cells at endpoint for each condition across independent experiments.

### Confocal imaging and colocalization

KPC cells were seeded in µ-Slide 8-well high slides (ibidi) using 3 × 10^3^ cells per well. Cells were allowed to attach and proliferate overnight. For endosomal localization experiments, cells were previously transfected in 6-cm plates using the corresponding plasmids and Lipofectamine 2000 (Thermo Fisher Scientific), and 24 h after transfection, cells were seeded into the chambered slides. The next day, cells were treated with either vehicle (PBS) or cET-ASOs accordingly and allowed to proliferate overnight (16 h). The next day, samples were fixed using a 4% paraformaldehyde solution in PBS and permeabilized with 0.1% Triton X-100 solution in PBS. Next, samples were incubated with blocking buffer (5% normal goat serum in TBS) for 1 h at RT. Primary antibodies were incubated overnight at 4°C, diluted in blocking buffer. The next day, samples were washed three times in TBS buffer and further incubated with the corresponding secondary antibodies and DAPI diluted in blocking buffer for 1 h at RT. Samples were next washed using TBS three times, and soft mounting medium (VECTASHIELD) was added to each well. Samples were visualized using either a Zeiss LSM880 microscope with Airyscan or a Zeiss Elyra 7 lattice SIM microscope, using a 63×/1.4NA objective and Zen Black software (v2.3 for Airyscan, 3.0 for Elyra). For Airyscan imaging, frame-sequential capture was used and channel-specific bandpass filters as follows: blue: 420–480 + LP605; green: 420–480 + 495–550; red: 420–480 + 495–620; far-red: 570–620 + LP645. Pixel size was 0.04 µm. Airyscan 3D processing was used at default sharpness. For SIM, lattices used were: for AlexaFluor488 G5 (27.5 µm); AlexaFluor568 G4 (32 µm) or G5 (27.5 µm) if phase modulation was judged to be sufficiently good; AlexaFluor647 G3 (36.5 µm); for DAPI G6 (23 µm) with 13 phases. z-stacks were captured at intervals of 91–125 nm depending on the experiment, or for some samples at 55 nm (over-sampling). Lasers (488, 561 and 642 nm, all 500 mW; 405 nm 50 mW) were typically used at 2.5–4% power. Emitted light was captured on PCO edge 4.2M sCMOS cameras via a Duolink adaptor, using a dual band-pass filter (490–560 + LP640) with a 50-ms exposure time. Processing was by SIM^2^ using “standard live” settings of input SNR medium, iterations 16, regularization 0.065; input and output sampling was set to ×4, median filter. Grating period was 718.33 nm, and the resulting xy scaling was 0.031 µm. Image analysis was performed using Fiji-ImageJ software to obtain Pearson correlation coefficients and fluorescence intensity profiles of representative vesicle examples. Fluorescence intensity profiles are shown as the fold change relative to the maximum intensity value for each fluorophore. For 3D image reconstruction, Imaris software was used to create the 3D surfaces using the SIM^2^ data generated after processing the images obtained using the Elyra7 microscope.

Opera Phenix (Revvity) and Harmony v5.2 software were used to acquire high-content confocal imaging. KPC cells were seeded on 96-well optical plates (Revvity Phenoplate, 5,000 cells/well). After 24 h, cells were treated with vehicle (PBS) or cET-ASOs as appropriate and allowed to proliferate overnight (for the 16-h time points). For the 4-h time points, cells were treated with vehicle (PBS) or cET-ASOs the following day. Vehicle and cET-ASOs were combined with bafilomycinA1 or chloroquine treatments at the appropriate concentrations. Cells were then fixed, permeabilized, and stained using the corresponding primary antibodies overnight at 4 °C. The following day, cells were incubated with DAPI and the corresponding fluorescent secondary antibodies at RT for 1 h. Cells were then imaged in an Opera Phenix (Revvity) high-throughput confocal microscope using either 20×/1.0NA water immersion (part HH14000421) or 63×/1.15NA water immersion (part HH14000423) objectives, and images were further analyzed using Harmony v5.2 software. For distance analysis, for each cell, vesicle distance to the nucleus was measured, and cells were binned into proximal groups (0–1 µm) and distal groups (from 1 µm to the maximum value). Then the mean fluorescence value for each particle was assessed for the corresponding markers such as EphA2, galectin-9, or ASO. Graphical representations and statistical analysis were performed using R version 4.5.0 within RStudio, running a custom pipeline for importing, harmonizing, and analyzing Opera Phenix/Harmony high-content imaging data. Data preprocessing relied on dplyr (1.1.4), readr (2.1.6), stringr (1.6.0), forcats (1.0.1), rlang (1.1.7), and tidyr. Visualization was performed using ggplot2 (4.0.1), ggridges (0.5.7), patchwork (1.3.2), and the HCL-based palette tools from colorspace (2.1–2). Interactive file selection and RStudio integration used rstudioapi (0.18.0). Statistical analysis modules used rstatix (0.7.3) for baseline tests, while more advanced models and contrasts relied on emmeans, lme4, lmerTest, and multcomp.

### Fluorescence polarization

These approaches were adapted from previously established protocols (Bhattacharya et al., 2017). 1 mg CD44 (Sino Biologicals) was dissolved in PBS to a concentration of 200 µM, from which a serial dilution was generated in PBS. For binding experiments, 50 nM Cy3-ASO or 1 μM Cy3-HA were incubated with increasing concentrations of CD44 (454 nM–50 μM) in PBS for 1 h at 30°C in a total volume of 10 μl. For competition experiments, 50 nM Cy3-ASO was incubated with 30 μM CD44 for 30 min at RT before increasing concentrations of unlabelled competitor HA (454 nM–50 μM) were added to the reaction. Fluorescence polarization of samples was then measured in a Tecan Spark (λ_ex_ = 535 nm; λ_em_ > 595 nm). Data were analyzed using GraphPad Prism (v10.5). To derive the dissociation constants, a 1:1 binding model was used to fit the data. Experiments were performed in triplicate.

### Proximity ligation

Proximity ligation was performed following the manufacturer’s (Merck Sigma-Aldrich) instructions. Briefly, 10,000 cells were seeded on an IBIDI 8-well chamber slide and allowed to attach overnight. The following day, cEt-ASOs were added to generate the 16-h time point and incubated overnight. 24 h later, cEt-ASOs were added to generate the 4-h time point. Cells were fixed in 4% paraformaldehyde for 15 min at RT and permeabilized using 0.3% Triton X-100 in PBS for 7 min. Cells were blocked using the blocking solution according to the manufacturer’s instructions, and the primary CD44-minus probe and the ASO antibody were mixed and incubated overnight at 4°C. Cells were incubated with the PLUS PLA probes in conjunction with Phalloidin-488 (1:400) and DAPI for 1 h at 37°C. Ligation and amplification protocols were then performed following the manufacturer’s instructions, and cells were prepared for imaging. Imaging was performed using a Zeiss LSM880 microscope incorporating Airyscan as detailed above. Images were analyzed using Fiji/ImageJ, and graphical representation and statistical analysis were performed using RStudio software as detailed above.

### High-throughput confocal live imaging

Opera Phenix (Revvity) and Harmony 5.2 software were used to acquire high-content confocal live imaging. KPC cells were seeded on 96-well optical plates (Revvity Phenoplate, 5,000 cells/well). 24 h later, cells were treated with vehicle (PBS) or cET-ASOs accordingly and allowed to proliferate overnight (16 h), in combination with either vehicle or liproxstatin-1 (1 μM). The next day, for the 4-h time points, cells were treated with vehicle (PBS) or cET-ASOs accordingly, as well as with either vehicle or liproxstatin-1. Cells were then stained with either CellROX green (5 μM) for total ROS quantification or C11-bodipy (5 μM) to assess LP, in combination with Hoechst 33342 to stain nuclei for 45 min at 37°C. Cells were then imaged in an Opera Phenix high-throughput confocal microscope using a 20×/1.0NA water immersion objective (part HH14000421) at 37°C and 5% CO_2_. Z-stacks were acquired and further analyzed using Harmony v5.2 software. Since CellROX green oxidation promotes its translocation to the nucleus and DNA binding (see the manufacturer’s CellROX Oxidative Stress Reagents manual), ROS production was measured as the ratio of CellROX detected in the nucleus versus the amount present in the cytoplasm. For C11-bodipy, LP was calculated as the fluorescence intensity of oxidized lipids (green, 488/510 nm) expressed as a ratio of that of reduced state lipids (red, 581/591 nm). Graphs and statistical analyses were performed using R (version 4.5.0) within RStudio, running a custom pipeline for importing, harmonizing, and analyzing Opera Phenix/Harmony high-content imaging data. Data preprocessing, visualization and statistical analysis modules were used as described above. High-resolution confocal images of KPC cells incubated with the C11-bodipy probe were obtained using a Zeiss LSM880 microscope comprising Airyscan and an incubation chamber at 37°C and 5% CO_2_.

### siRNA knockdown of SRs

H1299 cells were transfected with SR siRNA pools (see details in the “Reagents” section) using Nucleofection Kit V and an AMAXA nucleofector (Lonza). 1.5 × 10^5^ cells were then seeded onto 6-cm dishes and allowed to attach overnight. The next day, cells were incubated for 72 h with the corresponding doses of cEt-ASO. Cells were then lysed, and either protein or RNA was harvested. Effectiveness of siRNA knockdown as well as *KRAS* mRNA targeting and downstream signalling by cEt-ASOs were assessed by either western blotting or real-time PCR following the protocols described above.

### CRISPR knockout

Suppression of specific gene expression and generation of stable cell lines were achieved using the CRISPR-knockout genome editing system. Cloning of guide RNAs into lentiviral plasmids and generation of stable cell lines after lentiviral infection were performed according to the protocol established by the Zhang lab (Ran et al., 2013). Briefly, guide RNAs (see details in the “Reagents” section) were designed, annealed, and inserted into lentiCRISPRv2 lentiviral plasmids (#52961; Addgene). Lentiviral particles were produced by transducing HEK293FT cells with the lentiCRISPRv2 guide RNA–expressing plasmid together with psPAX2 and VSV-G packaging plasmids. Supernatants were collected after 48 h and transferred onto the recipient cells. Selection of positively transduced cells was assessed with antibiotic selection (puromycin) in the culture medium. After three passages, the successful knock-out of the target genes was assessed by qPCR as well as by western blotting.

### EPHA2-TurboID pulldown and immunofluorescence

KPC cells stably expressing an EPHA2-TurboID construct (Marco et al., 2021) were seeded in 10-cm plates using 2 × 10^6^ cells per plate. Cells were allowed to attach and proliferate overnight. The next day, cells were incubated with 100 µM biotin and 5 µM cET-ASO for 16 h. The next day, cells were placed on ice to stop the biotin ligation reaction and washed five times with ice-cold PBS. Cells were scraped from the plate in PBS and pelleted at 300×*g* for 3 min at 4°C. Cells were lysed in RIPA buffer supplemented with protease inhibitors and sonicated. The insoluble fraction was discarded, and the free biotin excess from the lysates was removed using an AMICON 3K filter column (Millipore). Cell lysates were recovered and further incubated with streptavidin-agarose beads (Millipore) for 2 h at 4°C. After the incubation, the beads were further washed sequentially with RIPA, 1M KCl, 0.1M Na_2_CO_3_, 2M urea, and a final 100 mM NaCl—10 mM Tris, pH 7.5—0.5 mM EDTA buffer. Beads were then resuspended in loading buffer and 2 mM biotin, and biotinylated targets were assessed using western blotting.

For immunofluorescence, KPC cells stably expressing an EPHA2-TurboID construct were plated onto glass-bottom 35-mm dishes and allowed to attach overnight. The next day, cells were treated with biotin as described above, and 16 h afterward, biotin was washed five times with PBS, and the cells were fixed using 4% paraformaldehyde. Cells were processed as described above, and biotinylated proteins were assessed using streptavidin conjugated to Alexa488 (Thermo Fisher Scientific). Samples were visualized in a Zeiss880 Airyscan microscope as described above.

### Genomic data

Publicly available data, analysis, and graphs were obtained from cBioPortal (Cerami et al., 2012) using the TCGA dataset corresponding to PAAD. GEPIA2 portal (Tang et al., 2019) was used to obtain the comparison between tumor and normal tissue data. Further genomic data regarding mRNA expression in PDAC were obtained from analysis of microarray and RNA-seq data by Bailey et al. (2016), as well as PDAC subtype classification expression data.

### Reagents

Reagents or resources are shown in Table 1.

**Table 1.** Reagent or resource

Reagent or resource	Source	Identifier
**Antibodies**	​	​
EPHA2 mouse WB 1:500 IF 1:100	SCBT	sc-398832
EPHA2 phospho Ser897 rabbit WB 1:1,000 IF 1:200	CST	#6347
EPHA2 phospho Tyr588 rabbit WB 1:1,000	CST	#12677
ASO rabbit IF 1:1,000	Ionis Pharma	Ionis 13545
MEK1/2 total rabbit WB 1:1,000	CST	#9126
MEK1/2 phospho Ser217/22 rabbit WB 1:1,000	CST	#9154
Vinculin mouse WB 1:1,000	SCBT	sc-73614
ERK1/2 total rabbit WB 1:1,000	Sigma-Aldrich	M5670
ERK1/2 phospho mouse WB 1:1,000	Sigma-Aldrich	M8159
KRAS mouse WB 1:1,000	LSBio	LS-C175665
p90RSK phospho S380 rabbit WB 1:1,000	CST	#9341
GAPDH mouse WB 1:1,000	SCBT	sc-365062
RPS6 phospho Ser235/236 rabbit WB 1:1,000	CST	#4858
CD44 rabbit WB 1:1,000 IF 1:100	CST	#37259
CD44-AF647 IF 1:500	BioLegend	103017
CD44 (for Duolink PLA)	BioLegend	103001
streptavidin 488 IF 1:400	Thermo Fisher Scientific	S11223
eIF2a phospho Ser51 rabbit WB 1:500	Abcam	ab32157
eIF2a mouse WB 1:1,000	CST	#2103
beta-Actin mouse WB 1:5,000	Sigma-Aldrich	AC-74
Histone H3 rabbit WB 1:1,000	CST	#4499
AKT phospho Ser473 rabbit WB 1:1,000	CST	#4060
JNK phospho Y16/T221 rabbit WB 1:1,000	CST	#9251
p38MAPK phospho T180/Y182 rabbit WB 1:1,000	CST	#4511
SCARB1 rabbit WB 1:1,000 IF 1:100	Proteintech	212277-1-AP
beta-Tubulin mouse WB 1:5,000	Sigma-Aldrich	T8328
G3BP1 rabbit WB 1:1,000	Proteintech	13057-2-AP
G3BP2 rabbit WB 1:1,000	Proteintech	16276-1-AP
eIF3b mouse IF 1:100	SCBT	sc-137214
IRDye 680RD Donkey anti-Mouse IgG Secondary Antibody donkey WB 1:5,000	Licor Bio	926-68072
IRDye 800CW Donkey anti-Rabbit IgG Secondary Antibody donkey WB 1:5,000	Licor Bio	926-32213
Anti-mouse IgG, HRP-linked Antibody horse WB 1:5,000	CST	#7076
Anti-rabbit IgG, HRP-linked Antibody goat WB 1:5,000	CST	#7074
Donkey anti-Mouse IgG (H+L) highly cross-Adsorbed Secondary Antibody, Alexa Fluor 488 donkey IF 1:400	Thermo Fisher Scientific	A21202
Donkey anti-Mouse IgG (H+L) highly cross-Adsorbed Secondary Antibody, Alexa Fluor 568 donkey IF 1:400	Thermo Fisher Scientific	A10037
Donkey anti-Mouse IgG (H+L) highly cross-Adsorbed Secondary Antibody, Alexa Fluor 647 donkey IF 1:400	Thermo Fisher Scientific	A31571
Donkey anti-Rabbit IgG (H+L) highly cross-Adsorbed Secondary Antibody, Alexa Fluor 488 donkey IF 1:400	Thermo Fisher Scientific	A21206
Donkey anti-Rabbit IgG (H+L) highly cross-Adsorbed Secondary Antibody, Alexa Fluor 568 donkey IF 1:400	Thermo Fisher Scientific	A10042
Donkey anti-Rabbit IgG (H+L) highly cross-Adsorbed Secondary Antibody, Alexa Fluor 647 donkey IF 1:400	Thermo Fisher Scientific	A31573
**Bacterial and virus strains**	​	​
One Shot Stbl3 Chemically Competent *E. coli*	Thermo Fisher Scientific	C737303
**Chemicals, peptides, and recombinant proteins**	​	​
Lipofectamine 2000	Thermo Fisher Scientific	11668027
Nucleofection Kit V	Lonza	VCA-1003
Quantinova SYBR Green RT-PCR Kit	Qiagen	208352
cET-ASO^KRAS^*Homo sapiens*	Ionis Pharma	​
cET-ASO^NT^	Ionis Pharma	​
RSKi – LJH685	selleckchem	S7870
Biotin	Sigma-Aldrich	B4501
Streptavidin-agarose beads	Millipore	16-126
Poly(dT) probe	Biosearch Technologies	Stellaris positive Control T30-CalFluor 590
ISRIB	MedchemExpress	HY-12495
BafilomycinA1	Cell Signaling	#54645
Chloroquine diphosphate salt	Merck Sigma-Aldrich	C6628
Recombinant human CD44 protein (ECD)	Sino Biologicals	12211-HNAH
Hyaluronate CyDye, MW 10 kDa	HAWORKS USA	HA-CyDye-10k
HA, MW 10 kDa	HAWORKS USA	HA-10k
Hoechst 33342	Thermo Fisher Scientific	H3570
CellROX green	Thermo Fisher Scientific	C10444
C11-bodipy 581/591	Thermo Fisher Scientific	D3861
Phalloidin-Alexa488	Thermo Fisher Scientific	A12379
**Commercial kits**	​	​
Duolink *In Situ* Detection Reagents Red	Merck Sigma-Aldrich	DUO92008
Duolink *In Situ* PLA probe Anti-Rabbit PLUS	Merck Sigma-Aldrich	DUO92002
Duolink *In Situ* Probemaker MINUS	Merck Sigma-Aldrich	DUO92010
Duolink *In Situ* Wash Buffers, Fluorescence	Merck Sigma-Aldrich	DUO82049
**Deposited data**	​	​
H1299 expression of SRrs - GSE179901	https://www.ncbi.nlm.nih.gov/geo/query/acc.cgi?acc=GSE179901	Data deposited in Marco et al. (2021)
**Experimental models: Cell lines**	​	​
NCI-H1299 cell line	ATCC	CRL-5803
HEK293FT	Thermo Fisher Scientific	R70007
KPC wild-type mouse tumor-derived cell line	Beatson Institute	As described in Gundry et al. (2017)
KPC *Epha2*^*−/−*^ mouse tumor-derived cell line	Beatson Institute	​
**Oligonucleotides (cET-ASOs)**	​	​
3-10-3 cEt for MALAT1 (mouse or human)	Ionis Pharmaceuticals	556089
3-10-3 cEt nontargeting ASO (mouse or human)	Ionis Pharmaceuticals	549148
3-10-3 cEt for human KRAS	Ionis Pharmaceuticals	651987 (a.k.a. AZD4785)
**Oligonucleotides (other)**	​	​
*Rab17 sgRNA #1* (5′-3′ sense/antisense) 5′-TCC​GAG​TGA​TGT​CAT​AAA​CC/GGT​TTA​TGA​CAT​CAC​TCG​GA-3′	Thermo Fisher Scientific	Custom 25 nM desalted primer
*Rab17 sgRNA #2* (5′-3′ sense/antisense) 5′-AGA​ACC​AGC​TTG​CTC​ACG​TA/TAC​GTG​AGC​AAG​CTG​GTT​CT-3′	Thermo Fisher Scientific	Custom 25 nM desalted primer
*Rab17 sgRNA #3* (5′-3′ sense/antisense) 5′-CCT​CAC​TTA​CAC​CCC​ACA​GT/ACT​GTG​GGG​TGT​AAG​TGA​GG-3′	Thermo Fisher Scientific	Custom 25 nM desalted primer
*Rab17 sgRNA #4* (5′-3′ sense/antisense) 5′-CAT​TGG​CAC​CCC​TGA​AGT​AG/CTA​CTT​CAG​GGG​TGC​CAA​TG-3′	Thermo Fisher Scientific	Custom 25 nM desalted primer
*Cd44 sgRNA #1* (5′-3′ sense/antisense) 5′-AAT​GTA​ACC​TGC​CGC​TAC​GC/GCG​TAG​CGG​CAG​GTT​ACA​TT-3′	Thermo Fisher Scientific	Custom 25 nM desalted primer
*Cd44 sgRNA #2* (5′-3′ sense/antisense) 5′-TGG​GTT​CAT​AGA​AGG​AAA​TG/CAT​TTC​CTT​CTA​TGA​ACC​CA-3′	Thermo Fisher Scientific	Custom 25 nM desalted primer
*Cd44 sgRNA #3* (5′-3′ sense/antisense) 5′-AGG​AGA​TCG​AGA​CTC​ATC​CA/TGG​ATG​AGT​CTC​GAT​CTC​CT-3′	Thermo Fisher Scientific	Custom 25 nM desalted primer
*Cd44 sgRNA #4* (5′-3′ sense/antisense) 5′-TAT​GGT​AAC​CGG​TCC​ATC​GA/TCG​ATG​GAC​CGG​TTA​CCA​TA-3′	Thermo Fisher Scientific	Custom 25 nM desalted primer
*Scarb1 sgRNA #1* (5′-3′ sense/antisense) 5′-GGG​GCC​GTG​AAG​CGA​TAC​GT/CGT​ATC​GCT​TCA​CGG​CCC​C-3′	Thermo Fisher Scientific	Custom 25 nM desalted primer
*Scarb1 sgRNA #2* (5′-3′ sense/antisense) 5′-GAG​GAT​TCG​GGT​GTC​ATG​AA/TTC​ATG​ACA​CCC​GAA​TCC​TC-3′	Thermo Fisher Scientific	Custom 25 nM desalted primer
*Scarb1 sgRNA #3* (5′-3′ sense/antisense) 5′-TCT​GAG​CCA​TGC​GAC​TTG​TC/GAC​AAG​TCG​CAT​GGC​TCA​GA-3′	Thermo Fisher Scientific	Custom 25 nM desalted primer
*Scarb1 sgRNA #4* (5′-3′ sense/antisense) 5′-GCT​GAT​GAT​GAC​CTT​GGC​GC/GCG​CCA​AGG​TCA​TCA​TCA​GC-3′	Thermo Fisher Scientific	Custom 25 nM desalted primer
*Epha2 sgRNA #1* (5′-3′ sense/antisense) 5′-GCT​GAC​CGT​GAT​CTC​GTC​AG/CTG​ACG​AGA​TCA​CGG​TCA​GC-3′	Thermo Fisher Scientific	Custom 25 nM desalted primer
*Epha2 sgRNA #2* (5′-3′ sense/antisense) 5′-TCG​GTG​TGC​AAC​GTG​GTA​TC/GAT​ACC​ACG​TTG​CAC​ACC​GA-3′	Thermo Fisher Scientific	Custom 25 nM desalted primer
*Epha2 sgRNA #3* (5′-3′ sense/antisense) 5′-GGT​CTA​TAA​AGG​GAC​GCT​GA/TCA​GCG​TCC​CTT​TAT​AGA​CC-3′	Thermo Fisher Scientific	Custom 25 nM desalted primer
*Epha2 sgRNA #4* (5′-3′ sense/antisense) 5′-TGT​CTC​CGA​TAC​ACA​ACC​CC/GGG​GTT​GTG​TAT​CGG​AGA​CA-3′	Thermo Fisher Scientific	Custom 25 nM desalted primer
*G3bp1 sgRNA #1* (5′-3′ sense/antisense) 5′-GCA​GTC​TAC​GGG​CAG​AAG​GT/ACC​TTC​TGC​CCG​TAG​ACT​GC-3′	Thermo Fisher Scientific	Custom 25 nM desalted primer
*G3bp1 sgRNA #2* (5′-3′ sense/antisense) 5′-AGG​CCC​CGG​ACA​TGT​TGC​AC/GTG​CAA​CAT​GTC​CGG​GGC​CT-3′	Thermo Fisher Scientific	Custom 25 nM desalted primer
*G3bp1 sgRNA #3* (5′-3′ sense/antisense) 5′-ACT​ACA​GAG​CAA​CAT​ACC​TC/GAG​GTA​TGT​TGC​TCT​GTA​GT-3′	Thermo Fisher Scientific	Custom 25 nM desalted primer
*G3bp1 sgRNA #4* (5′-3′ sense/antisense) 5′-TGT​GCA​GAA​GAG​CAC​TTC​CC/GGG​AAG​TGC​TCT​TCT​GCA​CA-3′	Thermo Fisher Scientific	Custom 25 nM desalted primer
*G3bp2 sgRNA #1* (5′-3′ sense/antisense) 5′-ATC​CAC​TCC​ACC​ATG​AAC​AT/ATG​TTC​ATG​GTG​GAG​TGG​AT-3′	Thermo Fisher Scientific	Custom 25 nM desalted primer
*G3bp2 sgRNA #2* (5′-3′ sense/antisense) 5′-GCT​AGC​TAG​CCT​TAC​GTC​AC/GTG​ACG​TAA​GGC​TAG​CTA​GC-3′	Thermo Fisher Scientific	Custom 25 nM desalted primer
*G3bp2 sgRNA #3* (5′-3′ sense/antisense) 5′-CCG​GAT​AGC​GAA​TTA​TTC​TC/GAG​AAT​AAT​TCG​CTA​TCC​GG-3′	Thermo Fisher Scientific	Custom 25 nM desalted primer
*G3bp2 sgRNA #4* (5′-3′ sense/antisense) 5′-TCT​GGC​TTA​GCA​TCA​ACC​CT/AGG​GTT​GAT​GCT​AAG​CCA​GA-3′	Thermo Fisher Scientific	Custom 25 nM desalted primer
*EPHA2 sgRNA #1* (5′-3′ sense/antisense) 5′-TCC​GTG​TGC​AAC​GTG​ATG​TC/GAC​ATC​ACG​TTG​CAC​ACG​GA-3′	Thermo Fisher Scientific	Custom 25 nM desalted primer
*EPHA2 sgRNA #2* (5′-3′ sense/antisense) 5′-GTG​AAG​GTG​TAG​TTC​ATG​TG/CAC​ATG​AAC​TAC​ACC​TTC​AC-3′	Thermo Fisher Scientific	Custom 25 nM desalted primer
*EPHA2 sgRNA #3* (5′-3′ sense/antisense) 5′-CAA​GTT​GCC​AGA​TCC​CTC​CG/CGG​AGG​GAT​CTG​GCA​ACT​TG-3′	Thermo Fisher Scientific	Custom 25 nM desalted primer
*EPHA2 sgRNA #4* (5′-3′ sense/antisense) 5′-CAC​ATA​TGA​GGA​CCC​CAA​CC/GGT​TGG​GGT​CCT​CAT​ATG​TG-3′	Thermo Fisher Scientific	Custom 25 nM desalted primer
*KRAS* real-time PCR primers (5′-3′ forward/reverse) 5′-ACA​GAG​AGT​GGA​GGA​TGC​TTT/TTT​CAC​ACA​GCC​AGG​AGT​CTT-3′	Thermo Fisher Scientific	Custom 25 nM desalted primer
*Kras* real-time PCR primers (5′-3′ forward/reverse) 5′-CAA​GAG​CGC​CTT​GAC​GAT​ACA​CCA​AGA​GAC​AGG​TTT​CTC​CAT​C-3′	Thermo Fisher Scientific	Custom 25 nM desalted primer
*18S* real-time PCR primers (5′-3′ forward/reverse) 5′-GCT​TAA​TTT​GAC​TCA​ACA​CGG​GAA​GCT​ATC​AAT​CTG​TCA​ATC​CTG​TC-3′	Thermo Fisher Scientific	Custom 25 nM desalted primer
*Msr1* real-time PCR primers (5′-3′ forward/reverse) 5′-GCA​CAA​TCT​GTG​ATG​ATC​GCT/CCC​AGC​ATC​TTC​TGA​ATG​TGA​A-3′	Thermo Fisher Scientific	Custom 25 nM desalted primer
*Colec12* real-time PCR primers (5′-3′ forward/reverse) 5′-GGT​GCA​GTC​CTT​CGG​TTA​CAA/GAT​GGT​CAG​TAA​GGC​ACA​CAG-3′	Thermo Fisher Scientific	Custom 25 nM desalted primer
*Scara5* real-time PCR primers (5′-3′ forward/reverse) 5′-CAT​GGA​TTT​CAC​AAT​GAT​TCG​CC/TCC​CCG​TCC​TTC​TTG​TCC​C-3′	Thermo Fisher Scientific	Custom 25 nM desalted primer
*Marco* real-time PCR primers (5′-3′ forward/reverse) 5′-ACA​GAG​CCG​ATT​TTG​ACC​AAG/CAG​CAG​TGC​AGT​ACC​TGC​C-3′	Thermo Fisher Scientific	Custom 25 nM desalted primer
*Scarb1* real-time PCR primers (5′-3′ forward/reverse) 5′-TTT​GGA​GTG​GTA​GTA​AAA​AGG​GC/TGA​CAT​CAG​GGA​CTC​AGA​GTA​G-3′	Thermo Fisher Scientific	Custom 25 nM desalted primer
*Cd36* real-time PCR primers (5′-3′ forward/reverse) 5′-AGA​TGA​CGT​GGC​AAA​GAA​CAG/CCT​TGG​CTA​GAT​AAC​GAA​CTC​TG-3′	Thermo Fisher Scientific	Custom 25 nM desalted primer
*Cd68* real-time PCR primers (5′-3′ forward/reverse) 5′-TGT​CTG​ATC​TTG​CTA​GGA​CCG/GAG​AGT​AAC​GGC​CTT​TTT​GTG​A-3′	Thermo Fisher Scientific	Custom 25 nM desalted primer
*Olr1* real-time PCR primers (5′-3′ forward/reverse) 5′-CAA​GAT​GAA​GCC​TGC​GAA​TGA/ACC​TGG​CGT​AAT​TGT​GTC​CAC-3′	Thermo Fisher Scientific	Custom 25 nM desalted primer
*Clec7a* real-time PCR primers (5′-3′ forward/reverse) 5′-GAC​TTC​AGC​ACT​CAA​GAC​ATC​C/TTG​TGT​CGC​CAA​AAT​GCT​AGG-3′	Thermo Fisher Scientific	Custom 25 nM desalted primer
*Mrc1* real-time PCR primers (5′-3′ forward/reverse) 5′-CTC​TGT​TCA​GCT​ATT​GGA​CGC/CGG​AAT​TTC​TGG​GAT​TCA​GCT​TC-3′	Thermo Fisher Scientific	Custom 25 nM desalted primer
*Asgr1* real-time PCR primers (5′-3′ forward/reverse) 5′-TGA​GCA​CCC​AGG​GAA​GTA​GT/CCA​TTG​CCC​CGA​AAT​GCA​G-3′	Thermo Fisher Scientific	Custom 25 nM desalted primer
*Scarf1* real-time PCR primers (5′-3′ forward/reverse) 5′-TGG​GAC​TAG​AGC​TGG​TGT​TCT/CAG​ATG​GGG​ATG​GTG​CAT​TCT-3′	Thermo Fisher Scientific	Custom 25 nM desalted primer
*Megf10* real-time PCR primers (5′-3′ forward/reverse) 5′-CCC​TCA​CTG​TGC​TGA​TAA​ATG​T/TGA​TGG​GGT​TAC​ACA​AAG​CTC-3′	Thermo Fisher Scientific	Custom 25 nM desalted primer
*Cxcl16* real-time PCR primers (5′-3′ forward/reverse) 5′-CCT​TGT​CTC​TTG​CGT​TCT​TCC/TCC​AAA​GTA​CCC​TGC​GGT​ATC-3′	Thermo Fisher Scientific	Custom 25 nM desalted primer
*Stab1* real-time PCR primers (5′-3′ forward/reverse) 5′-GGC​AGA​CGG​TAC​GGT​CTA​AAC/AGC​GGC​AGT​CCA​GAA​GTA​TCT-3′	Thermo Fisher Scientific	Custom 25 nM desalted primer
*Stab2* real-time PCR primers (5′-3′ forward/reverse) 5′-AGC​TGC​TGC​CTT​TAA​TCC​TCA/ACT​CCG​TCT​TGA​TGG​TTA​GAG​TA-3′	Thermo Fisher Scientific	Custom 25 nM desalted primer
*Cd163* real-time PCR primers (5′-3′ forward/reverse) 5′-ATG​GGT​GGA​CAC​AGA​ATG​GTT/CAG​GAG​CGT​TAG​TGA​CAG​CAG-3′	Thermo Fisher Scientific	Custom 25 nM desalted primer
*Cd163l1* real-time PCR primers (5′-3′ forward/reverse) 5′-CTG​GCC​TCT​GAG​TTT​AGG​GTC/CCC​TTG​GTG​TCG​AAC​CAG​C-3′	Thermo Fisher Scientific	Custom 25 nM desalted primer
*Mscart1* real-time PCR primers (5′-3′ forward/reverse) 5′-CCC​TTG​GTG​TCG​AAC​CAG​C/GTG​GCT​GTG​ATT​ACC​TGC​TCT-3′	Thermo Fisher Scientific	Custom 25 nM desalted primer
*Scart2* real-time PCR primers (5′-3′ forward/reverse) 5′-TTC​CCG​TTA​GTT​CCA​TCG​TGG/GGA​GAA​ATG​GCT​TTC​AAC​CTG​A-3′	Thermo Fisher Scientific	Custom 25 nM desalted primer
*Ager* real-time PCR primers (5′-3′ forward/reverse) 5′-CTT​GCT​CTA​TGG​GGA​GCT​GTA/GGA​GGA​TTT​GAG​CCA​CGC​T-3′	Thermo Fisher Scientific	Custom 25 nM desalted primer
*Cd44* real-time PCR primers (5′-3′ forward/reverse) 5′-TCG​ATT​TGA​ATG​TAA​CCT​GCC​G/CAG​TCC​GGG​AGA​TAC​TGT​AGC-3′	Thermo Fisher Scientific	Custom 25 nM desalted primer
*Lrp1* real-time PCR primers (5′-3′ forward/reverse) 5′-ACT​ATG​GAT​GCC​CCT​AAA​ACT​TG/GCA​ATC​TCT​TTC​ACC​GTC​ACA-3′	Thermo Fisher Scientific	Custom 25 nM desalted primer
*Lrp2* real-time PCR primers (5′-3′ forward/reverse) 5′-AAA​ATG​GAA​ACG​GGG​TGA​CTT/GGC​TGC​ATA​CAT​TGG​GTT​TTC​A-3′	Thermo Fisher Scientific	Custom 25 nM desalted primer
**siRNA**	​	​
CD44	Horizon Discovery	L-009999-00-0005
SCARB1	Horizon Discovery	L-010592-00-0005
LRP1	Horizon Discovery	L-004721-00-0005

Recombinant DNA	​
RAB17 - RAB17-mCherry	As described by Diaz-Vera et al. (2017)
EPHA2^WT^ - EPHA2 WT-GFP	As described by Gundry et al. (2017)
EPHA2^S897A^ - EPHA2 phospho-defective S897-GFP	​
EPHA2^NLS^ - EPHA2 K583A K586A-GFP	As described by Marco et al. (2021)
EPHA2-turboID - EPHA2 WT-TurboID-T2A-BFP *H*. *sapiens*	​
Galectin-9 - Galectin-9-mCherry	Addgene #166689
G3BP1 - G3BP1-GFP	From Gerald McInerney, Karolinska Institute, as described by Panas et al. (2015)
RAB11 – Rab11-mCherry	As described by Gundry et al. (2017)
RAB14 – Rab14-mCherry	​
RCP – RCP-mCherry	​
**Software and algorithms**	​
Zeiss Zen (black edition) system 2.3	Carl Zeiss Microscopy
Zeiss Zen (blue edition) 2.3	Carl Zeiss Microscopy
Imaris 10.1.1	Oxford Instruments
ImageJ – Fiji 2.6.0	As described by Schindelin et al. (2012)
GraphPad Prism 10.3.1	GraphPad Software LLC
CFX Maestro Software for CFX Real-Time PCR 2.3	Bio-Rad
Image Studio Software 6.0	Li-Cor
Harmony High-Content Imaging and Analysis Software 4.9	Revvity
Columbus Image Data Storage and Analysis System 2.9.1	Revvity
R version 4.5.0 (2025-04-11 ucrt)	Posit Software, PBC

### Online supplemental material

Fig. S1 shows supplementary data relates to main Figs. 1 and 2. Fig. S1, A–H inclusive, presents data pertaining to human PDAC supporting main Fig. 1, A–C. Fig. S1, I–Q inclusive, presents data supporting the analysis of the influence of cET-ASO^*Kras*^ on KPC and H1299 cells. Fig. S2 shows supplementary data relates to main Fig. 3 and presents data supporting the analysis of SR function and the signalling downstream of these receptors in ASO uptake. Fig. S3 shows supplementary data relates to main Fig. 5 and to the more discursive, final section of the results and discussion. Fig. S3, A–M inclusive, presents data supporting the analysis of endosome leakiness and the role of SG components in endosomal repair covered in Fig. 5. Fig. S3 N presents a schematic summary depicting the endocytic pathway transporting ASOs from the extracellular space into the perinuclear region and enabling their release from nuclear-captured leaky endosomes.

## Supplementary Material

Review History

SourceData F1 is the source file for Fig. 1.

SourceData F2 is the source file for Fig. 2.

SourceData F3 is the source file for Fig. 3.

SourceData F5 is the source file for Fig. 5.

SourceData FS1 is the source file for Fig. S1.

SourceData FS2 is the source file for Fig. S2.

SourceData FS3 is the source file for Fig. S3.

## Data Availability

The data supporting the findings of this study are available within the article and its supplementary information files and from the corresponding author upon request.
