# Supplementary material for: EPHA2/CD44-directed trafficking enhances endosomal leakiness and antisense therapy delivery
Source: J Cell Biol. 2026 Aug 11;225(9):e202507217. doi: 10.1083/jcb.202507217 (PMC13521514; doi:10.1083/jcb.202507217)
Supplement: Supplementary file 1 [file jcb_202507217_review_history.pdf]

EPHA2/CD44-directed trafficking enhances endosomal leakiness and antisense therapy delivery

Sergi Marco, Peter Walsh, Alexey Revenko, Tobias Schmidt, Peter Thomason, Lynn McGarry, A. Robert MacLeod, Sonam Ansel, Dina Tataran, Martin Bushell, Chiara Braconi, and Jim Norman

Corresponding Author(s): Jim Norman, Cancer Research UK Scotland Institute and Sergi Marco, Cancer Research UK Scotland Institute

**Review Timeline:**		
	Submission Date:	2025-07-31
	Editorial Decision:	2025-09-11
	Revision Received:	2026-03-25
	Editorial Decision:	2026-04-15
	Revision Received:	2026-05-28

Monitoring Editor: Pier Paolo Di Fiore

Scientific Editor: Dan Simon

Transaction Report:

(Note: With the exception of the correction of typographical or spelling errors that could be a source of ambiguity, letters and reports are not edited. The original formatting of letters and referee reports may not be reflected in this compilation.)

DOI: https://doi.org/10.1083/jcb.202507217

September 11, 2025

Re: JCB manuscript #202507217

Jim Norman
Cancer Research UK Scotland Institute

Dear Prof. Norman,

Thank you for submitting your manuscript entitled "EPHA2/CD44-directed trafficking enhances endosomal leakiness and antisense therapy delivery." The manuscript was assessed by expert reviewers, whose comments are appended to this letter. We invite you to submit a revision if you can address the reviewers' key concerns, as outlined here.

You will see that a main question raised by both reviewers is whether cET-ASOs directly binds to CD44 which we agree should be addressed experimentally. Reviewer #1 also asks for an extended characterization of endosomal leakiness which should also be done, although we feel that analysis of the spatiotemporal dynamics of leakage is not essential. The other comments can be addressed with text revisions.

While you are revising your manuscript, please also attend to the following editorial points to help expedite the publication of your manuscript. Please direct any editorial questions to the journal office.

GENERAL GUIDELINES:
Text limits: Character count for a Report is < 20,000, not including spaces. Count includes title page, abstract, introduction, the joint Results & Discussion, and acknowledgments. Count does not include materials and methods, figure legends, references, tables, or supplemental legends.

Figures: Reports may have up to 5 main text figures. To avoid delays in production, figures must be prepared according to the policies outlined in our Instructions to Authors, under Data Presentation, https://jcb.rupress.org/site/misc/ifora.xhtml. All figures in accepted manuscripts will be screened prior to publication.

IMPORTANT: It is JCB policy that if requested, original data images must be made available. Failure to provide original images upon request will result in unavoidable delays in publication. Please ensure that you have access to all original microscopy and blot data images before submitting your revision.

Supplemental information: There are strict limits on the allowable amount of supplemental data. Reports may have up to 3 supplemental figures. Up to 10 supplemental videos or flash animations are allowed. A summary of all supplemental material should appear at the end of the Materials and methods section.

Please note that JCB now requires authors to submit Source Data used to generate figures containing gels and Western blots with all revised manuscripts. This Source Data consists of fully uncropped and unprocessed images for each gel/blot displayed in the main and supplemental figures. For assays performed using capillary electrophoresis and/or immunoassay-based detection, authors should instead provide the electropherogram graph(s) for each experiment, plotting fluorescence/chemiluminescence intensity vs. molecular weight/size. Please be sure to provide one Source Data file for each figure gels, blots, and/or capillary electrophoresis assays along with your revised manuscript files. File names for Source Data figures should be alphanumeric without any spaces or special characters (i.e., SourceDataF#, where F# refers to the associated main figure number or SourceDataFS# for those associated with Supplementary figures). For traditional gels and blots, the lanes of the gels/blots should be labeled as they are in the associated figure, the place where cropping was applied should be marked (with a box), and molecular weight/size standards should be labeled wherever possible. For capillary electrophoresis assays, each trace in the graph should be color-coded and labeled to indicate which protein, gene, or sample is being measured (please try to avoid red/green combinations to accommodate our color-blind readers).

Source Data files will be made available to reviewers during evaluation of revised manuscripts and, if your paper is eventually published in JCB, the files will be directly linked to specific figures in the published article.

Source Data Figures should be provided as individual PDF files (one file per figure). Authors should endeavor to retain a minimum resolution of 300 dpi or pixels per inch. Please review our instructions for export from Photoshop, Illustrator, and PowerPoint here: https://rupress.org/jcb/pages/submission-guidelines#revised

The typical timeframe for revisions is three to four months. If you anticipate any difficulties in meeting this aforementioned revision time limit, please contact us and we can work with you to find an appropriate time frame for resubmission. Please note that papers are generally considered through only one revision cycle, so any revised manuscript will likely be either accepted or rejected.

When submitting the revision, please include a cover letter addressing the reviewers' comments point by point. Please also highlight all changes in the text of the manuscript.

We hope that the comments below will prove constructive as your work progresses. We would be happy to discuss them further once you've had a chance to consider the points raised in this letter.

Thank you for this interesting contribution to Journal of Cell Biology. You can contact us at the journal office with any questions at cellbio@rockefeller.edu.

Sincerely,

Pier Paolo Di Fiore, MD, PhD
Monitoring Editor
Journal of Cell Biology

Dan Simon, PhD
Scientific Editor
Journal of Cell Biology

Reviewer #1 (Comments to the Authors (Required)):

The manuscript by Marco et al. presents a comprehensive study identifying an endocytic pathway that governs the productive uptake and intracellular efficacy of therapeutic antisense oligonucleotides (ASOs). The work provides a framework for ASO delivery and suggests a new strategy to potentiate nucleic acid-based therapies in cancers that overexpress EPHA2 and CD44.

While this is a significant study, several points should be addressed to strengthen the conclusions and solidify the proposed model:

1-Regarding the ASO-CD44 Interaction: Is the binding of the polyanionic ASO to CD44 direct, and if so, what is its affinity? More importantly, is the activation of the downstream MAPK/p90RSK pathway a specific consequence of CET-ASO binding, or is it a general response to the binding of any polyanionic ligand to CD44?

2-On the Generality of the Pathway: The study convincingly demonstrates this pathway for a KRAS-targeting CET-ASO in cancer models with high EPHA2 expression. However, the broader applicability of these findings needs to be addressed. Is this trafficking route specific to the cET chemistry, or is it a general mechanism for other ASO modifications or even other nucleic acid therapeutics like siRNAs? Furthermore, is this pathway exclusively active in cancer cells, or does it also operate in non-transformed cells expressing EPHA2 and CD44, which would have implications for potential off-target effects?

3-On the Mechanism of Leakage: Are ASO-containing endosomes that are not near the nucleus also leaky? Since leakage occurs in a certain percentage of lysosomes, do the authors have any evidence for the specific mechanism leading to this leakage? Alternatively, do the authors propose that docking at the nuclear pore induces membrane damage? If so, how?

4-Regarding the Role of pH: Could the authors use pH-sensitive probes or available ratiometric reporters to provide insight into the potential role of pH in the observed leakage? For instance, are lysosomes of a certain pH more vulnerable to damage?

5-On the Spatiotemporal Dynamics of Leakage: Could the authors quantify the percentage of leaky lysosomes at different time points and determine if there is any spatial relationship to their location (e.g., nuclear proximity vs. peripheral localization)?

6-Regarding Lysosome Repair Machinery: The authors show that GAL-9 co-localizes with the stress granule marker G3BP1. Do the authors identify other lysosome repair markers, such as ESCRT-III proteins, PITT pathway proteins, or ANXA1/2/7? A previous study has shown that lysosome repair is deficient in G3BP DKO cells despite the recruitment of other membrane repair proteins (10.1038/s41586-023-06726-w), which suggests a predominant role for condensates as membrane-stabilizing agents upstream of the repair machinery. Do the authors observe if these other markers are still recruited to leaky lysosomes in the G3BP DKO cells?

7-On the Consequences of Inhibiting Endosomal Repair: While inhibiting the stress granule (SG) pathway appears to be a promising strategy to enhance ASO efficacy, have the authors assessed whether this approach leads to unintended consequences, such as increased inflammation or cell death?

8-Regarding In Vivo Validation: While likely beyond the scope of the current study, showing that the efficacy of the KRAS-targeting ASO is diminished in Epha2-knockout tumors in a syngeneic mouse model would provide powerful validation for the

entire pathway.

Reviewer #2 (Comments to the Authors (Required)):

The manuscript addresses an important problem in the field of RNA therapeutics and presents interesting data that could advance our understanding of ASO optimization as it pertains to its uptake, escape and suppression of its target mRNA in cells.
Summary of main points:
1. EPHA2 is required for productive uptake of KRAS-targeting ASOs: The authors showed uptake in both EPHA2 positive and negative cells. This shows the effect of EPHA2 on the process, but not EPHA2 specifically binding and internalizing the ASO. The authors did show that CD44 was involved, though its specific binding with the ASO is not demonstrated. Despite that, these molecules certainly seem to be involved with internalization of ASO and subsequent trafficking. Also see point #2 below.
2. EPHA2-mediated nuclear-capture is required for cET-ASOKras to suppress KRAS expression. EPHA2 has a NLS and that cargo is adjacent to the nucleus for engagement. Fig. 5 is a good illustration of the process. They also show that Rab17 is involved for vesicle trafficking and Rab17 KO cells do not suppress KRAS expression very well which means the cargo is not arriving at the destination.
3. Scavenger receptors trigger EPHA2-dependent uptake and trafficking of ASOs. The authors demonstrate phosphorylation of Ser897 occurs upon ASO uptake and signalling proceeds through p90RSK but not AKT. An inhibitor for p90RSK, LHJ685, abrogates this effect. This is also where they detect CD44 in close proximity with EPHA2 and use the BioID tagging to identify it as a possible receptor for uptake. There are indicators that CD44 is involved with uptake, but the paper falls short on definitively proving that this is a receptor for binding/internalization of ASO. See point #2 below.
4. EPHA2 is required for cET-ASO trafficking to leaky endosomes. Cargo escapes the endosomes by leakage which is detected by Gal9 and G3BP1. The authors inhibit the stress granule assembly which results in increased suppression of KRAS expression (more escape of cargo) without affecting endocytosis.

All statistics and reference data are in order. Overall, very nice paper and very informative. There are just a few minor issues that need to be addressed.

1. Need a detailed schematic and source of the ASO used for these experiments.
2. The first section "EPHA2 is required for productive uptake of KRAS-targeting ASOs" describes cells are EPHA2+/+ and EPHA2-/- affects ASO uptake and KRAS targeting. If EPHA2 is a receptor that binds cET-ASOs, there should be some data to show this. The data in this experiment merely shows that endocytosis may be a secondary effect and that EPHA2 may be more involved with stabilization of the endosome. Fig 1E shows overlap in fluorescent signaling but this alone does not prove that it is a binding receptor, just a receptor in the vicinity of the ASO. The use of the BioID approach is novel which resulted in the identification of CD44. CD44 is intimately tied in with the underlying actin cytoskeleton and is not an endocytic receptor but goes along for the ride when there is increased membrane ruffling. Mechanistically, the authors did a fine job with showing that EPHA2 is phosphorylated on Ser897, but is all of this in response to increased membrane ruffling as opposed to receptor signaling for classical endocytosis? This may be partially answered by a paper by Odaka et al (PMID:38855323).
3. Line 437: ON should be written out as "overnight"
4. The SR screening methods should be included in the methods section. Fig. 3E
5. There are a few grammatical errors throughout the manuscript so the revision should be carefully read.

Response to reviewers

Marco et al.

EPHA2/CD44-directed trafficking enhances endosomal leakiness and antisense therapy delivery.

****

Reviewer 1

We would like to thank this reviewer for their positive view of the manuscript and for their constructive criticisms/concerns which we have addressed with the inclusion of new data.

Points

1. Regarding the ASO-CD44 Interaction: Is the binding of the polyanionic ASO to CD44 direct, and if so, what is its affinity? More importantly, is the activation of the downstream MAPK/p90RSK pathway a specific consequence of CET-ASO binding, or is it a general response to the binding of any polyanionic ligand to CD44?

We have addressed this as follows:

We have performed additional experimentation using two complementary approaches – fluorescence polarisation spectroscopy and proximity ligation microscopy – to evaluate potential interactions between ASO and CD44. Both these methods indicate that cET-ASO and CD44 interact directly with one another and we have included these data in the revision of the paper as follows:

a) Fluorescence polarisation spectroscopy indicates direct intermolecular binding between cET-ASO and CD44:

   a. New figure: Fig. 3H,I
   b. Manuscript: Page 6, line 212 "We considered whether CD44 and cET-ASOs can directly associate to form a receptor-ligand complex. We used fluorescence polarisation spectroscopy to evaluate any association between purified CD44 and Cy3-labelled cET-ASO, and compared this with CD44's ability to associate with its canonical cognate ligand, hyaluronic acid (HA; average molecular ≈10 kDa) conjugated to Cy3. This indicated that Cy3-labelled cET-ASO and HA both associated with CD44 with micromolar affinity (displaying apparent K_Ds of 10.8 and 1.8 µM respectively) (Fig. 3H). Moreover, association of Cy3-labelled cET-ASO with CD44 was displaced by incubation with unlabelled HA (Fig. 3I), indicating that ASOs and HA likely compete for binding site(s) within CD44."

b) ***Proximity ligation approach indicates close proximity of cET-ASO and CD44 in cells:***

   a. New figure: Fig. 3J,K
   b. Manuscript: Page 6, line 221 "We next used proximity ligation to determine whether CD44 and cET-ASOs associate *in situ*. We incubated KPC cells with cET-ASO^{Kras} for 4 or 16 hr, then visualised these using antibodies recognising cET-ASO and CD44 conjugated to proximity ligation probes. This indicated that cET-ASO^{Kras} and CD44 come into close proximity (<40nm) in cells, reinforcing that these two molecules associate directly with one another (Fig. 3J,K)."

Additionally, on this point, to address whether *"the activation of the downstream MAPK/p90RSK pathway a specific consequence of CET-ASO binding, or is it a general response to the binding of any polyanionic ligand to CD44?"* we have compared the ability of cET-ASOs and short chain hyaluronic acid (HA) to activate signalling downstream of CD44. This indicated that addition of short chain HA (10kDa/25-mer oligo at either 1 or 5µM) promotes signalling downstream of CD44, as evidenced by increased EphA2 Ser^{897}, ERK1/2 and p90RSK phosphorylation, and does so to a similar extent as

observed following addition of cET-ASO (5µM) (see reviewer Fig. R1 below). These data indicate that binding of polyanionic ligands to CD44 can activate the downstream MAPK/p90RSK signalling.

Reviewer figure R1

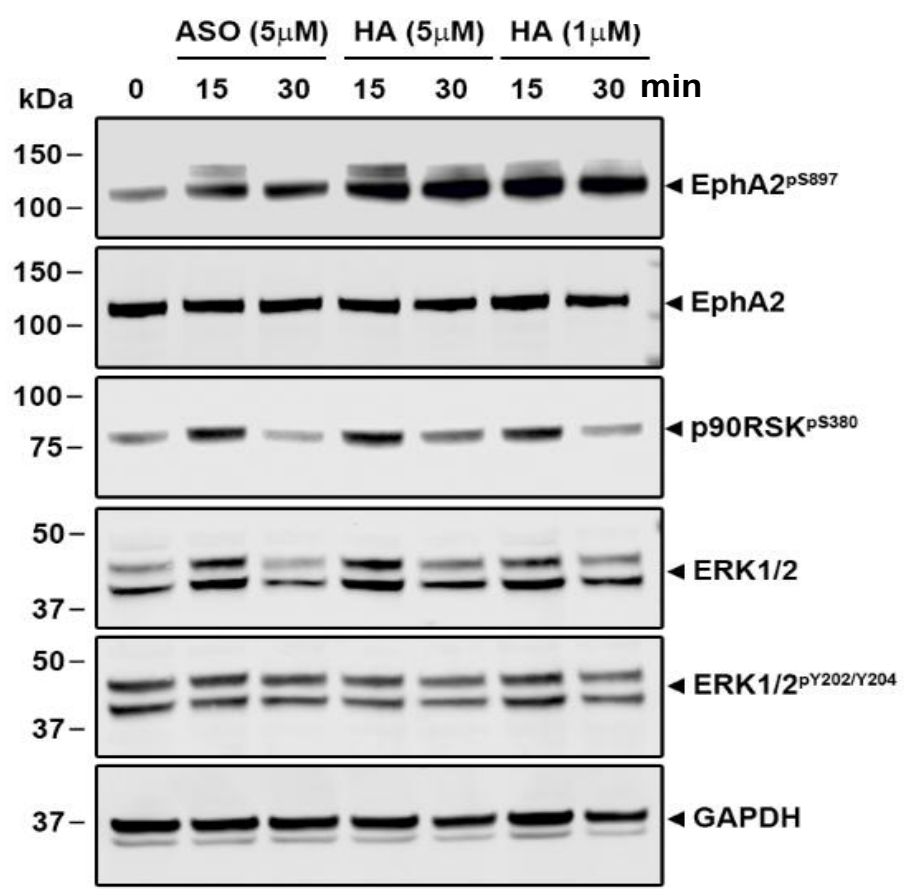

Fig. R1. cET-ASO and HA can drive signalling downstream of CD44. KPC cells were incubated with cET-ASO^{NT} or hyaluronic acid (HA; 10kDa) at the indicated concentrations for 15 or 30 min. Cells were lysed and levels of phosphorylated and total EPHA2 Ser^{897}, p90RSK, and ERK1/2 determined using Western blot with GAPDH as loading control.

2. On the Generality of the Pathway: The study convincingly demonstrates this pathway for a KRAS-targeting CET-ASO in cancer models with high EPHA2 expression. However, the broader applicability of these findings needs to be addressed. Is this trafficking route specific to the cET chemistry, or is it a general mechanism for other ASO modifications or even other nucleic acid therapeutics like siRNAs? Furthermore, is this pathway exclusively active in cancer cells, or does it also operate in non-transformed cells expressing EPHA2 and CD44, which would have implications for potential off-target effects?

We have addressed this as follows:
We have attempted to measure the internalisation of certain siRNAs in the KPC pancreatic cancer and H1299 lung cancer cells that we have employed for this study. However, the results from these experiments have been ambiguous. Our interim conclusion from this is that cET-ASO may be restricted to the pathway that we describe in this paper, whereas siRNAs have more freedom to follow other pathways into the cell. However, as we are unable to draw firm conclusions on this matter, we concede that these studies are outside the scope of the current manuscript.

3. On the Mechanism of Leakage: Are ASO-containing endosomes that are not near the nucleus also leaky? Since leakage occurs in a certain percentage of lysosomes, do the authors have any evidence for the specific mechanism leading to this leakage? Alternatively, do the authors propose that docking at the nuclear pore induces membrane damage? If so, how?

We have addressed this as follows:
We have investigated this in two ways. Firstly (see a) below), we have compared the recruitment of galectin-9 (marker for leaky endosomes) to cET-ASO-containing endosomes in the nuclear vicinity (nuclear proximal vesicles) with those cET-ASO-containing vesicles which are more distant (nuclear distal vesicles) from the nucleus, and assessed how this is influenced by expression of mutants of EphA2 which are defective in nuclear-capture (EphA2^{NLS}). This indicated that leaky ASO-containing

endosomes are concentrated in the nuclear vicinity, and that this is strongly dependent in expression of EphA2's ability to mediate their nuclear-capture. Secondly (see b) below), we now report that reactive oxygen species (ROS) generation and lipid peroxidation likely form part of the mechanism leading to endosomal leakage and have included these data in our revision of the paper.

a) ***Role of nuclear docking on leakage***
Figures: Fig. 2B, Fig. 5C-F
Manuscript:
Page 4, line 139 "…endocytosed cET-ASO^{Kras} was detectable in a nuclear-proximal population of EphA2-positive endosomal vesicles 4hr following addition of the ASO to KPC cells (Fig. 2A, B)" and page 8, line 281 "…cells expressing a nuclear-capture defective EphA2 mutant (*EphA2*^{NLS}) showed significantly reduced colocalisation of cET-ASOs with both EphA2 and galectin-9 (Fig. 5C,D). Notably, colocalisation of both EphA2 (Fig. 5E) and galectin-9 (Fig. 5F) with ASO in *EphA2*^{NLS}-expressing cells was particularly strongly reduced in nuclear-proximal vesicles, indicating that EphA2 nuclear-capture is crucial for the correct trafficking of ASOs to endosomes with the potential to become leaky."

b) ***ROS production and peroxidation of endosomal lipids is driven by cET-ASO addition***
New figures: Fig. 4A-E
Manuscript: Page 7, line 261 "…to interrogate whether ASO uptake may evoke oxygen radical-mediated endomembrane damage we treated KPC cells with cET-ASO^{Kras} and measured nuclear translocation of the CellROX green probe which detects intracellular ROS species. This indicated that cET-ASO^{Kras} addition significantly increased cellular ROS levels (Fig. 4A,B). Since lipid peroxidation (LP) is thought to be a principal mechanism driving ROS-mediated endosomal membrane damage, we used the C11-Bodipy probe to detect oxidised lipids. This indicated that addition of cET-ASO^{Kras} to KPC cells increased levels of LP (Fig. 4C,D) and this was observed to occur in intracellular vesicles (Fig. 4E). Notably, the ASO-induced increases in both ROS and LP were opposed by addition of the radical-trapping antioxidant liproxstatin-1 (Fig. 4A,D)."

4. Regarding the Role of pH: Could the authors use pH-sensitive probes or available ratiometric reporters to provide insight into the potential role of pH in the observed leakage? For instance, are lysosomes of a certain pH more vulnerable to damage?

We have addressed this as follows:
Assuming that the 'internal' pH of a leaky compartment would be similar to that of the cytosol, we reasoned that it would be necessary to measure the luminal pH of compartments that were nuclear-captured prior to their becoming leaky to determine the influence of pH on the vulnerability to damage. To overcome the considerable technical challenges of trying to pinpoint what is likely to be a transient state (captured but not yet leaky) using pH-sensitive probes, we investigated the consequences of using agents that reduce endosomal acidification on ASO trafficking to nuclear-captured vesicles, and on the recruitment of galectin-9 to ASO-containing vesicles. These experiments indicate that blockade of endosome acidification reduces trafficking of cET-ASO to nuclear-captured endosomes and opposes recruitment of galectin-9 suggesting that endosomes cannot rendered leaky by ASO addition when endosomal maturation is disrupted.

a) ***Effect of bafilomycin and chloroquine on cET-ASO trafficking***
Figures: Fig. 2L, Fig. S1Q
Manuscript: Page 5, line 164 "…addition of either bafilomycinA1 (Fig. 2L) or chloroquine (Fig. S1Q) significantly reduced accumulation of ASOs within endosomal vesicles at both 4hr and 16hr following ASO addition to KPC cells, indicating that endosomal acidification is required for appropriate intracellular trafficking of ASOs to nuclear-proximal vesicle populations."

b) ***Effect of bafilomycin on galectin-9 recruitment to ASO-containing vesicles***
New figures: Fig. S3C
Manuscript: Page 8, line 280 "…addition of bafilomycinA1, to oppose endosomal acidification/maturation, significantly reduced recruitment of galectin-9 to ASO-positive vesicles (Fig. S3C), suggesting that endosomes cannot rendered leaky by ASO addition when endosomal maturation is disrupted"

Finally on this point, as this reviewer recommends, we used a pH-sensitive probe to measure the pH of intracellular vesicles following cET-ASO addition. To do this we treated cells with cET-ASO (at 0.5 or 5 μM) for 4 or 16 hr, and then added pHrodo green, a fluorescence probe that responds to pH; the lower the pH (acid) the higher the green fluorescence of the probe. This indicated that addition of cET-ASO increases vesicular pH in a concentration-dependent manner, whereas a control internalisation cargo (dextran) did not do this (see reviewer Fig. R2 below). One interpretation of this observation is that cET-ASOs oppose endosome acidification by promoting leakiness, thus highlighting the difficulties with trying to determine how acidification might increase vulnerability to membrane compartment damage.

Reviewer figure R2

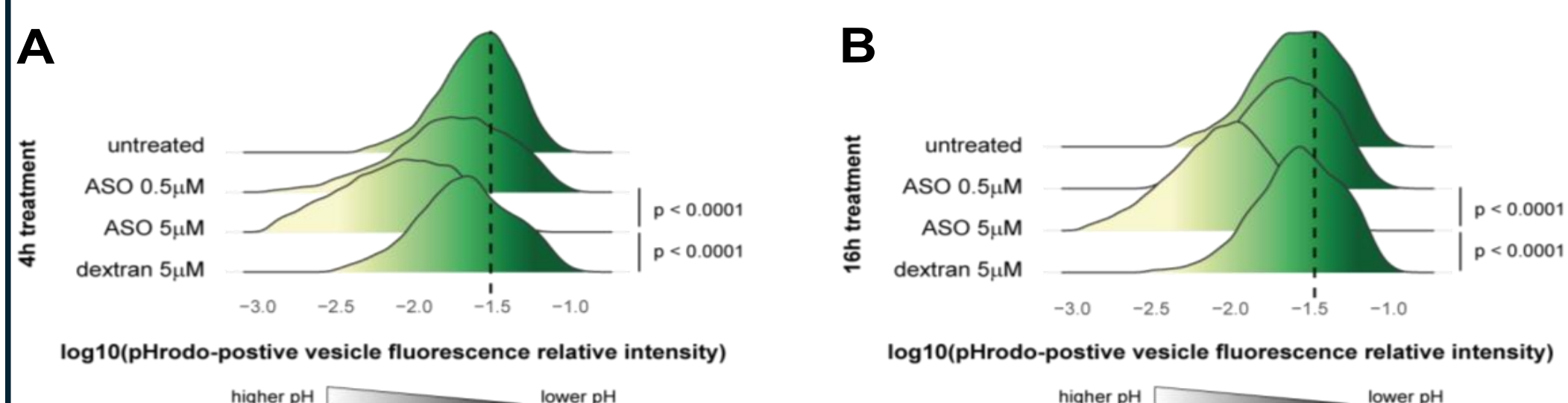

Fig. R2. Addition of cET-ASOs increases the pH of intracellular vesicles. KPC cells were incubated with cET-ASO^{NT} or dextran at the indicated concentrations for 4 (A) or 16 (B) hours. Cells were then incubated with pHrodo green and imaged. Vesicular fluorescence was quantified; the lower the pH (acid) the greater the intensity of the probe.

5. On the Spatiotemporal Dynamics of Leakage: Could the authors quantify the percentage of leaky lysosomes at different time points and determine if there is any spatial relationship to their location (e.g., nuclear proximity vs. peripheral localization)?

We have addressed this as follows:
In addressing this reviewer's previous point concerning the role of nuclear-capture in leakiness we have measured the relationship between galectin-9 recruitment to ASO-contain vesicles and their distance from the nucleus (see above; reviewer 1, point 3). We have, however, not further investigated the spatiotemporal dynamics of acquisition of endosomal leakiness as we feel that this lies outwith the scope of the current manuscript. Indeed, the editors commented in their decision letter that "….analysis of the spatiotemporal dynamics of leakage is not essential".

6. Regarding Lysosome Repair Machinery: The authors show that GAL-9 co-localizes with the stress granule marker G3BP1. Do the authors identify other lysosome repair markers, such as ESCRT-III proteins, PITT pathway proteins, or ANXA1/2/7? A previous study has shown that lysosome repair is deficient in G3BP DKO cells despite the recruitment of other membrane repair proteins (10.1038/s41586-023-06726-w), which suggests a predominant role for condensates as membrane-stabilizing agents upstream of the repair machinery. Do the authors observe if these other markers are still recruited to leaky lysosomes in the G3BP DKO cells?

We have addressed this as follows:
We have investigated the localisation of representatives of the ESCRT-III complex, (CHMP4B & VPS4B), PITT pathway proteins (ATG2)) and annexins (ANXA1, ANXA2 & ANXA7) with respect to internalised labelled cET-ASO. From these studies we have been unable to demonstrate any convincing recruitment of components of the ESCRT-III complex, annexins, or PITT pathway proteins to cET-ASO-containing endosomes in G3BP DKO cells (see reviewer Fig. R3 below for examples of staining for CMMP4B, ANXA2 & ANXA7 whereas VPS4B and ATG2 are not shown). This supports a view that stress granule-type condensates may play a predominant role in membrane repair either upstream of or independently from canonical membrane repair machinery.

Reviewer figure R3

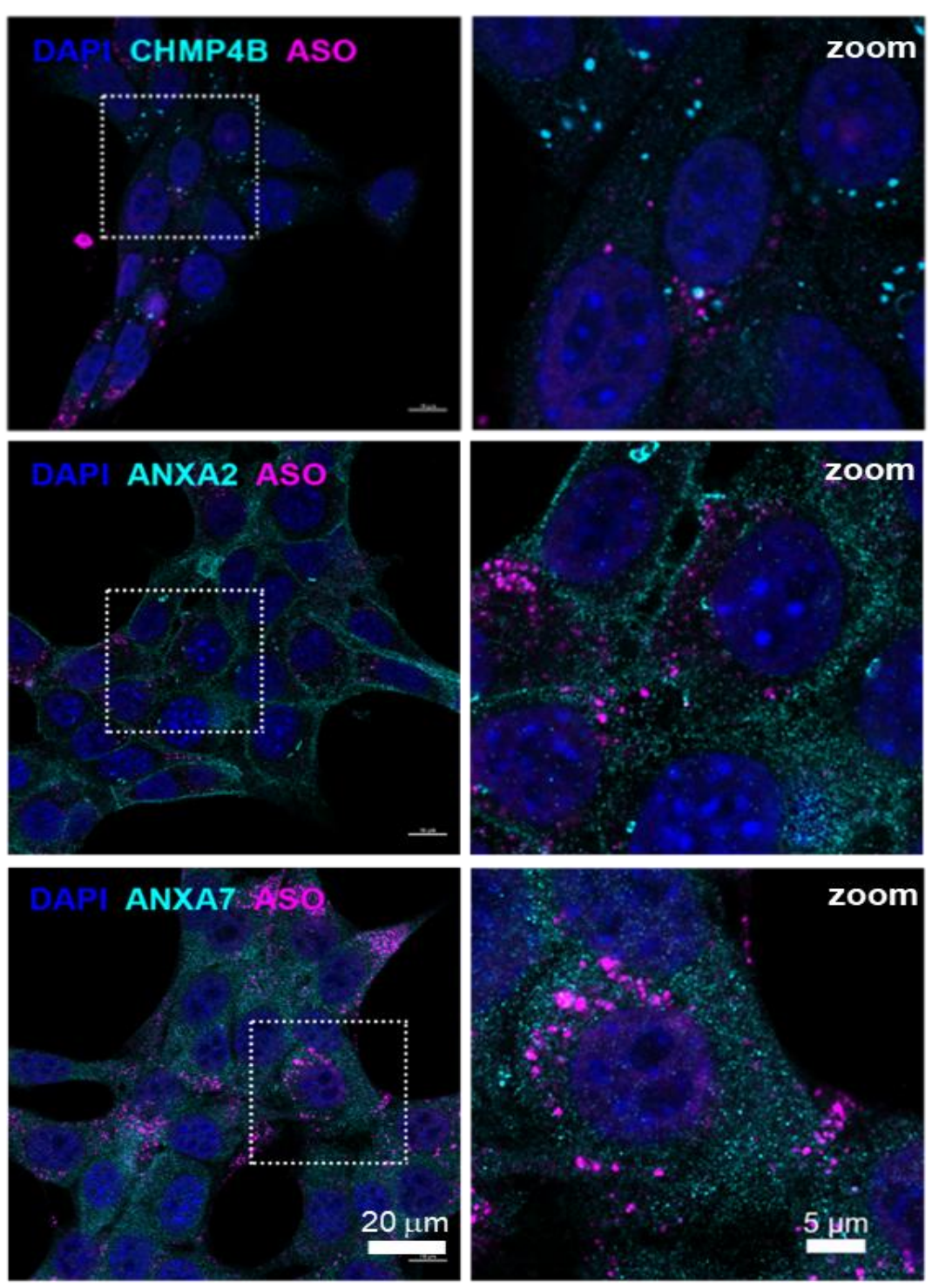

Fig. R3. CHMP4B and annexins-2&7 do not colocalise with internalised cET-ASOs. G3BP DKO KPC cells were incubated with Cy3-labelled cET-ASO^{NT} for 16hr and fixed. CVHMP4B, ANXA2 and ANXA7 were then visualised using immunofluorescence followed by confocal microscopy. CHMP4B and annexin and staining is displayed in cyan, cET-ASO^{NT} is displayed in magenta and DAPI-stained nuclei are blue.

7. On the Consequences of Inhibiting Endosomal Repair: While inhibiting the stress granule (SG) pathway appears to be a promising strategy to enhance ASO efficacy, have the authors assessed whether this approach leads to unintended consequences, such as increased inflammation or cell death?

Reply:
Whilst we have not addressed this reviewer comment directly, we consider it probable that opposing endomembrane repair may influence levels of ROS production and Fe^{3+}-catalysed lipid peroxidation.

Both would be expected to influence signalling leading to increased inflammatory cytokine production and ferroptotic cell death. We will certainly consider these possibilities in future studies, but consider this invest4igation to be outwith the scope of the present manuscript.

8. Regarding In Vivo Validation: While likely beyond the scope of the current study, showing that the efficacy of the KRAS-targeting ASO is diminished in Epha2-knockout tumors in a syngeneic mouse model would provide powerful validation for the entire pathway.

Reply:
It is certainly our intention to test the efficacy targeting tumour growth and aggressiveness (invasion and metastasis) with cET-ASO in KRAS-driven autochthonous mouse models of metastatic carcinoma – such as the KPC model of PDAC, and KRAS-driven models of lung and colon adenocarcinoma. Moreover, we are strategising experiments in which we will assess the efficacy of ISRIB as an adjunct agent to increase productive cET-ASO uptake in vivo. However, these experiments will take a considerable amount of time and, as such, we consider them to be outwith the scope of this short report.

Reviewer 2

We would like to thank this reviewer for their positive view of the manuscript and for their constructive criticisms/concerns which we have addressed with the inclusion of new data. Also, we find this reviewer's summary statements and interpretation of our paper to be erudite and apposite.

Main Points

1. EPHA2 is required for productive uptake of KRAS-targeting ASOs: The authors showed uptake in both EPHA2 positive and negative cells. This shows the effect of EPHA2 on the process, but not EPHA2 specifically binding and internalizing the ASO. The authors did show that CD44 was involved, though its specific binding with the ASO is not demonstrated. Despite that, these molecules certainly seem to be involved with internalization of ASO and subsequent trafficking. Also see point #2 below.

We have addressed this as follows (reply is as for reviewer 1, comment 1):

We have performed additional experimentation using two complementary approaches – fluorescence polarisation spectroscopy and proximity ligation microscopy – to evaluate potential interactions between ASO and CD44. Both these methods indicate that cET-ASO and CD44 interact directly with one another and we have included these data in the revision of the paper as follows:

a) ***Fluorescence polarisation spectroscopy indicates direct intermolecular binding between cET-ASO and CD44:***
    a. New figure: Fig. 3H,I
    b. Manuscript: Page 6, line 212 "We considered whether CD44 and cET-ASOs can directly associate to form a receptor-ligand complex. We used fluorescence polarisation spectroscopy to evaluate any association between purified CD44 and Cy3-labelled cET-ASO, and compared this with CD44's ability to associate with its canonical cognate ligand, hyaluronic acid (HA; average molecular ≈10 kDa) conjugated to Cy3. This indicated that Cy3-labelled cET-ASO and HA both associated with CD44 with micromolar affinity (displaying apparent K_Ds of 10.8 and 1.8 μM respectively) (Fig. 3H). Moreover, association of Cy3-labelled cET-ASO with CD44 was displaced by incubation with unlabelled HA (Fig. 3I), indicating that ASOs and HA likely compete for binding site(s) within CD44."

b) ***Proximity ligation approach indicates close proximity of cET-ASO and CD44 in cells:***
    a. New figure: Fig. 3J,K
    b. Manuscript: Page 6, line 221 "We next used proximity ligation to determine whether CD44 and cET-ASOs associate *in situ*. We incubated KPC cells with cET-ASO^{Kras} for 4 or 16 hr, then visualised these using antibodies recognising cET-ASO and CD44 conjugated to proximity ligation probes. This indicated that cET-ASO^{Kras} and CD44 come into close proximity (<40nm) in cells, reinforcing that these two molecules associate directly with one another (Fig. 3J,K)."

2. EPHA2-mediated nuclear-capture is required for cET-ASOKras to suppress KRAS expression. EPHA2 has a NLS and that cargo is adjacent to the nucleus for engagement. Fig. 5 is a good illustration of the process. They also show that Rab17 is involved for vesicle trafficking and Rab17 KO cells do not suppress KRAS expression very well which means the cargo is not arriving at the destination.

Reply:

Yes, this is indeed how we interpret our data. We have now added experimentation to flesh-out this mechanism a little more, including the provision of evidence that the nuclear-capture process encourages endosomal leakiness and that compartment acidification is required for trafficking to the juxtanuclear region and for the acquisition of compartment leakiness.

3. Scavenger receptors trigger EPHA2-dependent uptake and trafficking of ASOs. The authors demonstrate phosphorylation of Ser897 occurs upon ASO uptake and signalling proceeds through p90RSK but not AKT. An inhibitor for p90RSK, LHJ685, abrogates this effect. This is also where they detect CD44 in close proximity with EPHA2 and use the BioID tagging to identify it as a possible receptor for uptake. There are indicators that CD44 is involved with uptake, but the paper falls short on definitively proving that this is a receptor for binding/internalization of ASO. See point #2 below.

Reply:
Please refer to our reply to this reviewer's comment 1 above and to reviewer 1's comment 1. In these replies we describe how we have demonstrated direct association between CD44 and cET-ASOs using fluorescence polarisation spectroscopy and proximity ligation fluorescence microscopy.

4. EPHA2 is required for cET-ASO trafficking to leaky endosomes. Cargo escapes the endosomes by leakage which is detected by Gal9 and G3BP1. The authors inhibit the stress granule assembly which results in increased suppression of KRAS expression (more escape of cargo) without affecting endocytosis.

Reply:
Yes, we concur with this view and interpretation of our data.

Minor Points

1. Need a detailed schematic and source of the ASO used for these experiments.
Reply:
We have detailed the source of the cET-ASOs in the STAR reagents table in the methods section.

2. The first section "EPHA2 is required for productive uptake of KRAS-targeting ASOs" describes cells are EPHA2+/+ and EPHA2-/- affects ASO uptake and KRAS targeting. If EPHA2 is a receptor that binds cET-ASOs, there should be some data to show this. The data in this experiment merely shows that endocytosis may be a secondary effect and that EPHA2 may be more involved with stabilization of the endosome. Fig 1E shows overlap in fluorescent signaling but this alone does not prove that it is a binding receptor, just a receptor in the vicinity of the ASO. The use of the BioID approach is novel which resulted in the identification of CD44. CD44 is intimately tied in with the underlying actin cytoskeleton and is not an endocytic receptor but goes along for the ride when there is increased membrane ruffling. Mechanistically, the authors did a fine job with showing that EPHA2 is phosphorylated on Ser897, but is all of this in response to increased membrane ruffling as opposed to receptor signaling for classical endocytosis? This may be partially answered by a paper by Odaka et al (PMID:38855323).

Reply:
We feel that this comment has been addressed by our demonstration of direct interaction between CD44 and cET-ASO (see above).

3. Line 437: ON should be written out as "overnight"4. The SR screening methods should be included in the methods section. Fig. 3E5. There are a few grammatical errors throughout the manuscript so the revision should be carefully read.

Reply:
Thank you!! This has been corrected.

April 15, 2026

RE: JCB Manuscript #202507217R

Jim Norman
Cancer Research UK Scotland Institute

Dear Prof. Norman,

Thank you for submitting your revised manuscript entitled "EPHA2/CD44-directed trafficking enhances endosomal leakiness and antisense therapy delivery" which has been re-assessed by the two original reviewers. We would be happy to publish your paper in JCB pending final revisions necessary to meet our formatting guidelines (see details below). Please also address the important final comment of Reviewer #2

To avoid unnecessary delays in the acceptance and publication of your paper, please read the following information carefully.

A. MANUSCRIPT ORGANIZATION AND FORMATTING:

Full guidelines are available on our Instructions for Authors page, https://jcb.rupress.org/submission-guidelines#revised.
Submission of a paper that does not conform to JCB guidelines will delay the acceptance of your manuscript.

1) Text limits: Character count for Reports is < 20,000, not including spaces. Count includes title page, abstract, introduction, results & discussion, and acknowledgments. Count does not include materials and methods, figure legends, references, tables, or supplemental legends.
Reports must have a single 'Results and Discussion' section.

2) Figure formatting: Reports may have up to 5 main text figures. Scale bars must be present on all microscopy images, including inset magnifications. Molecular weight or nucleic acid size markers must be included on all gel electrophoresis.

Also, please avoid pairing red and green for images and graphs to ensure legibility for color-blind readers. If red and green are paired for images, please ensure that the particular red and green hues used in micrographs are distinctive with any of the colorblind types. If not, please modify colors accordingly or provide separate images of the individual channels.

3) Statistical analysis: Error bars on graphic representations of numerical data must be clearly described in the figure legend. The number of independent data points (n) represented in a graph must be indicated in the legend. Please indicate whether 'n' refers to technical or biological replicates (i.e. number of analyzed cells, samples or animals, number of independent experiments). If independent experiments with multiple biological replicates have been performed, we recommend using distribution-reproducibility SuperPlots (please see Lord et al., JCB 2020) to better display the distribution of the entire dataset, and report statistics (such as means, error bars, and P values) that address the reproducibility of the findings.

Statistical methods should be explained in full in the materials and methods. For figures presenting pooled data the statistical measure should be defined in the figure legends. Please also be sure to indicate the statistical tests used in each of your experiments (both in the figure legend itself and in a separate methods section) as well as the parameters of the test (for example, if you ran a t-test, please indicate if it was one- or two-sided, etc.). Also, if you used parametric tests, please indicate if the data distribution was tested for normality (and if so, how). If not, you must state something to the effect that "Data distribution was assumed to be normal but this was not formally tested."

4) Materials and methods: Should be comprehensive and not simply reference a previous publication for details on how an experiment was performed. Please provide full descriptions (at least in brief) in the text for readers who may not have access to referenced manuscripts. The text should not refer to methods "...as previously described." Please also indicate the type of membrane used for immunoblotting as well as describe acquisition and quantification methods.

5) For all cell lines, vectors, strains, constructs/cDNAs, etc. - all genetic material: please include database / vendor ID (e.g. Addgene, ATCC, etc.) or if unavailable, please briefly describe their basic genetic features, even if described in other published work or gifted to you by other investigators (and provide references where appropriate). Please be sure to provide the sequences for all of your oligos: primers, si/shRNA, RNAi, gRNAs, etc. in the materials and methods. You must also indicate in the methods the source, species, and catalog numbers/vendor identifiers (where appropriate) for all of your antibodies, including secondary. If antibodies are not commercial, please add a reference citation if possible.

6) Microscope image acquisition: The following information must be provided about the acquisition and processing of images:
a. Make and model of microscope
b. Type, magnification, and numerical aperture of the objective lenses

c. Temperature
d. Imaging medium
e. Fluorochromes
f. Camera make and model
g. Acquisition software
h. Any software used for image processing subsequent to data acquisition. Please include details and types of operations involved (e.g., type of deconvolution, 3D reconstitutions, surface or volume rendering, gamma adjustments, etc.).

7) References: There is no limit to the number of references cited in a manuscript. References should be cited parenthetically in the text/tables by author and year of publication. Abbreviate the names of journals according to PubMed.

8) Supplemental materials: Reports may have up to 3 supplemental figures and 10 videos. Please also note that tables, like figures, should be provided as individual, editable files. A summary of all supplemental material should appear at the end of the Materials and methods section. Please include one brief sentence per item.

9) eTOC summary: A ~40-50 word summary that describes the context and significance of the findings for a general readership should be included on the title page. The statement should be written in the present tense and refer to the work in the third person. It should begin with "First author name(s) et al..." to match our preferred style.

10) Conflict of interest statement: JCB requires inclusion of a statement in the acknowledgements regarding competing financial interests. If no competing financial interests exist, please include the following statement: "The authors declare no competing financial interests." If competing interests are declared, please follow your statement of these competing interests with the following statement: "The authors declare no further competing financial interests."

11) A separate author contribution section is required following the Acknowledgments in all research manuscripts. All authors should be mentioned and designated by their first and middle initials and full surnames. We encourage use of the CRediT nomenclature (https://casrai.org/credit/).

12) ORCID IDs: ORCID IDs are unique identifiers allowing researchers to create a record of their various scholarly contributions in a single place. Please note that ORCID IDs are required for all authors. At resubmission of your final files, please be sure to provide your ORCID ID and those of all co-authors.

13) JCB requires authors to submit Source Data used to generate figures containing gels and Western blots with all revised manuscripts. This Source Data consists of fully uncropped and unprocessed images for each gel/blot displayed in the main and supplemental figures. For assays performed using capillary electrophoresis and/or immunoassay-based detection, authors should instead provide the electropherogram graph(s) for each experiment, plotting fluorescence/chemiluminescence intensity vs. molecular weight/size. Since your paper includes cropped gel and/or blot images, please be sure to provide one Source Data file for each figure gels, blots, and/or capillary electrophoresis assays along with your revised manuscript files. File names for Source Data figures should be alphanumeric without any spaces or special characters (i.e., SourceDataF#, where F# refers to the associated main figure number or SourceDataFS# for those associated with Supplementary figures). For traditional gels and blots, the lanes of the gels/blots should be labeled as they are in the associated figure, the place where cropping was applied should be marked (with a box), and molecular weight/size standards should be labeled wherever possible. For capillary electrophoresis assays, each trace in the graph should be color-coded and labeled to indicate which protein, gene, or sample is being measured (please try to avoid red/green combinations to accommodate our color-blind readers).

Source Data files will be directly linked to specific figures in the published article. Source Data Figures should be provided as individual PDF files (one file per figure). Authors should endeavor to retain a minimum resolution of 300 dpi or pixels per inch. Please review our instructions for export from Photoshop, Illustrator, and PowerPoint here: https://rupress.org/jcb/pages/submission-guidelines#revised.

14) Journal of Cell Biology now requires a data availability statement for all research article submissions. These statements will be published in the article directly above the Acknowledgments. The statement should address all data underlying the research presented in the manuscript. Please visit the JCB instructions for authors for guidelines and examples of statements at (https://rupress.org/jcb/pages/editorial-policies#data-availability-statement).

B. FINAL FILES:

Please upload the following materials to our online submission system. These items are required prior to acceptance. If you have any questions, contact JCB's Managing Editor, Lindsey Hollander (lhollander@rockefeller.edu).

-- An editable version of the final text (.DOC or .DOCX) is needed for copyediting (no PDFs).

-- High-resolution figure and MP4 video files: See our detailed guidelines for preparing your production-ready images, https://jcb.rupress.org/fig-vid-guidelines.

-- Cover images: If you have any striking images related to this story, we would be happy to consider them for inclusion on the journal cover. Submitted images may also be chosen for highlighting on the journal table of contents or JCB homepage carousel. Images should be uploaded as TIFF or EPS files and must be at least 300 dpi resolution.

It is JCB policy that if requested, original data images must be made available to the editors. Failure to provide original images upon request will result in unavoidable delays in publication. Please ensure that you have access to all original data images prior to final submission.

The license to publish form must be signed before your manuscript can be sent to production. A link to the license to publish form will be sent to the corresponding author only. Please take a moment to check your funder requirements before choosing the appropriate license.

Additionally, JCB encourages authors to submit a short video summary of their work. These videos are intended to convey the main messages of the study to a non-specialist, scientific audience. Think of them as an extended version of your abstract, or a short poster presentation. We encourage first authors to present the results to increase their visibility. The videos will be shared on social media to promote your work. For more detailed guidelines and tips on preparing your video, please visit https://rupress.org/jcb/pages/submission-guidelines#videoSummaries.

Thank you for your attention to these final processing requirements. Please revise and format the manuscript and upload materials within 14 days. If you need an extension for whatever reason, please let us know and we can work with you to determine a suitable revision period.

Please contact the journal office with any questions at cellbio@rockefeller.edu.

Thank you for this interesting contribution, we look forward to publishing your paper in Journal of Cell Biology.

Sincerely,

Pier Paolo Di Fiore, MD, PhD
Editor
Journal of Cell Biology

Dan Simon, PhD
Scientific Editor
Journal of Cell Biology

Reviewer #1 (Comments to the Authors (Required)):

The authors have successfully addressed all of my comments with several new experiments that have strengthened this interesting study.

Reviewer #2 (Comments to the Authors (Required)):

The authors addressed my major concerns with this paper by adding several more experiments (fluorescence polarization and proximity ligation) which were well-done. One of my minor points was not addressed sufficiently and that was the chemical schematic of the ASO used in the manuscript. They identified the source as Ionis Pharma, however, Ionis makes a lot of ASOs and there are many variants of the cET-ASOKras that differ by chemistry, not by targeting sequence. All their ASOs are cataloged by a unique identifying number. These numbers at the very least should be added to the table in the methods section or it would be impossible to repeat/confirm any of these experiments without monumental effort.

Response to reviewers

Marco et al.

EPHA2/CD44-directed trafficking enhances endosomal leakiness and antisense therapy delivery.

****

Reviewer 2

Points

1. The authors addressed my major concerns with this paper by adding several more experiments (fluorescence polarization and proximity ligation) which were well-done. One of my minor points was not addressed sufficiently and that was the chemical schematic of the ASO used in the manuscript. They identified the source as Ionis Pharma, however, Ionis makes a lot of ASOs and there are many variants of the cET-ASOKras that differ by chemistry, not by targeting sequence. All their ASOs are cataloged by a unique identifying number. These numbers at the very least should be added to the table in the methods section or it would be impossible to repeat/confirm any of these experiments without monumental effort.

We have addressed this as follows:

We have now added this information to the 'reagents' subsection of the Materials and Methods. Specifically, we have added a category now entitled 'Oligonucleotides (cET-ASOs)'. In this we have provided the six figure unique identifying numbers for the Ionis Pharmaceutical ASOs that we have used in the study.
