# Supplementary material for: EPHA2/CD44-directed trafficking enhances endosomal leakiness and antisense therapy delivery
Source: J Cell Biol. 2026 Aug 11;225(9):e202507217. doi: 10.1083/jcb.202507217 (PMC13521514; doi:10.1083/jcb.202507217)

FIGURE 2D

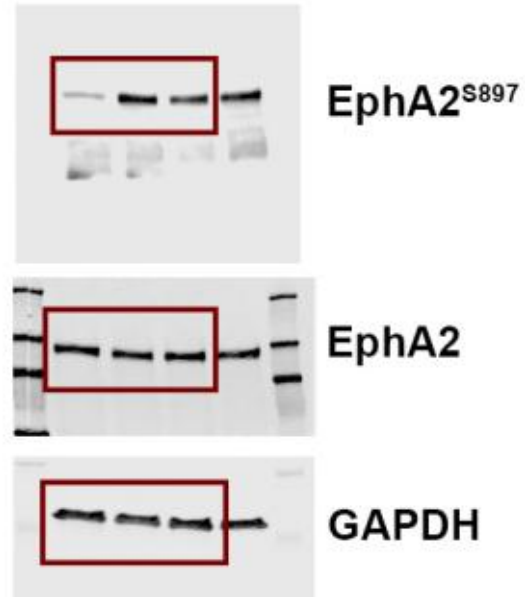

FIGURE 2H

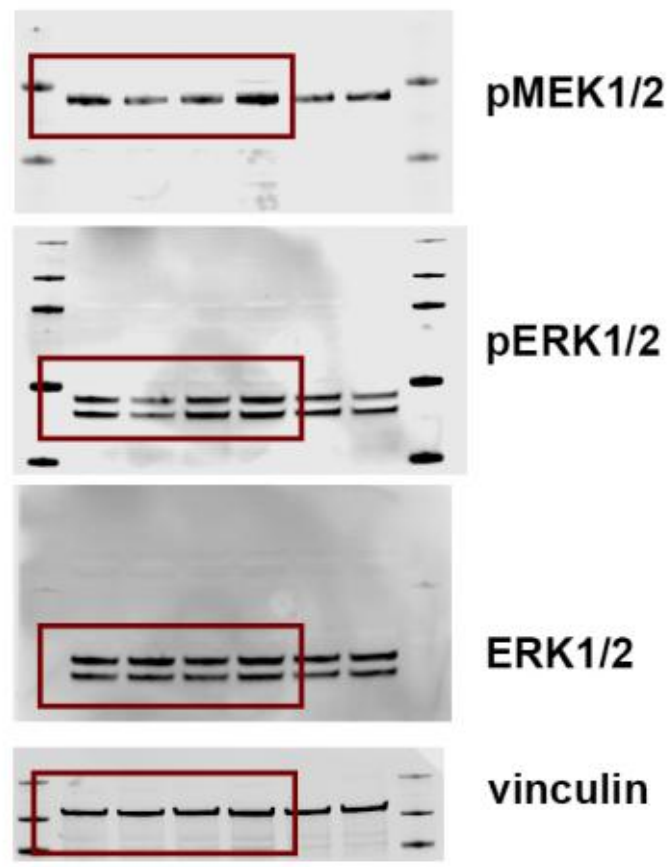

FIGURE 2J

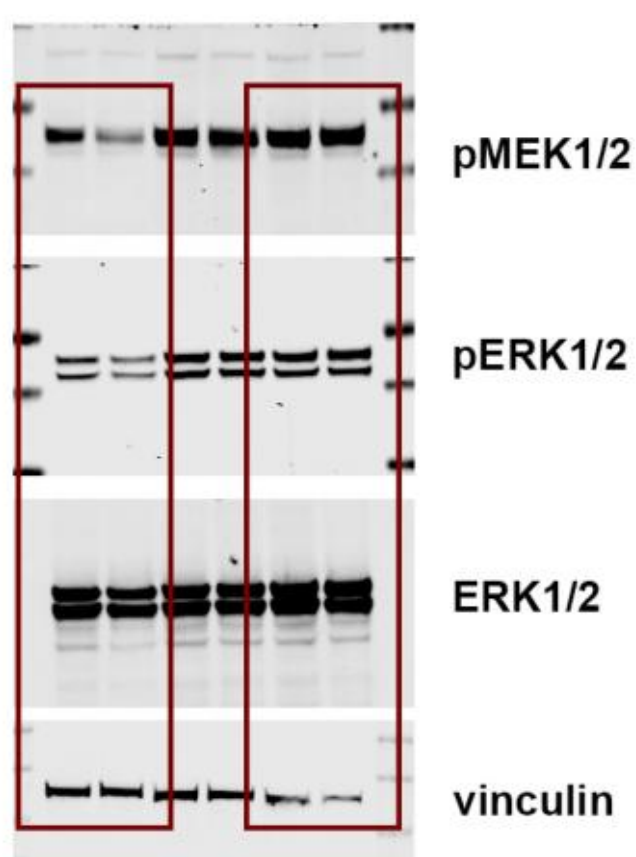

Supplement: Supplementary file 3 [file jcb_202507217_sourcedataf2.pdf]
