# Supplementary material for: EPHA2/CD44-directed trafficking enhances endosomal leakiness and antisense therapy delivery
Source: J Cell Biol. 2026 Aug 11;225(9):e202507217. doi: 10.1083/jcb.202507217 (PMC13521514; doi:10.1083/jcb.202507217)

FIGURE 3A

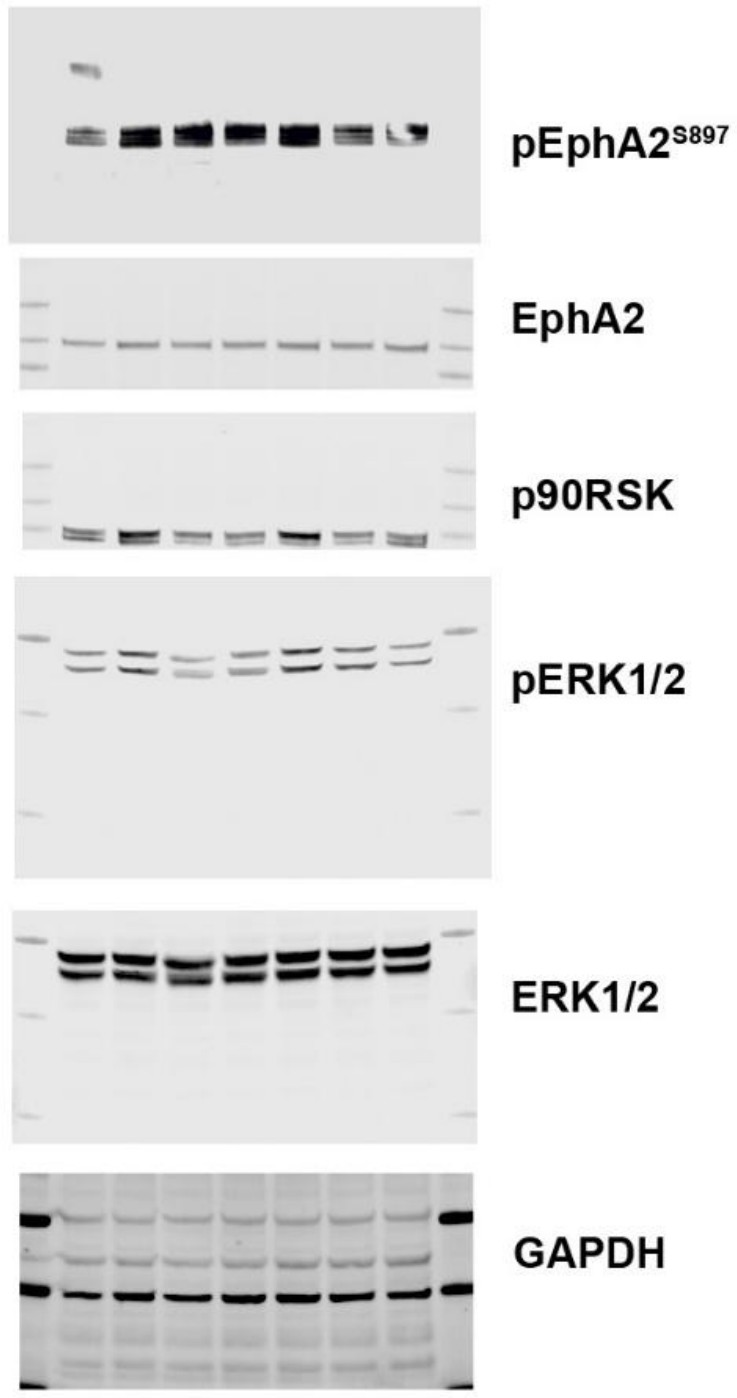

FIGURE 3B

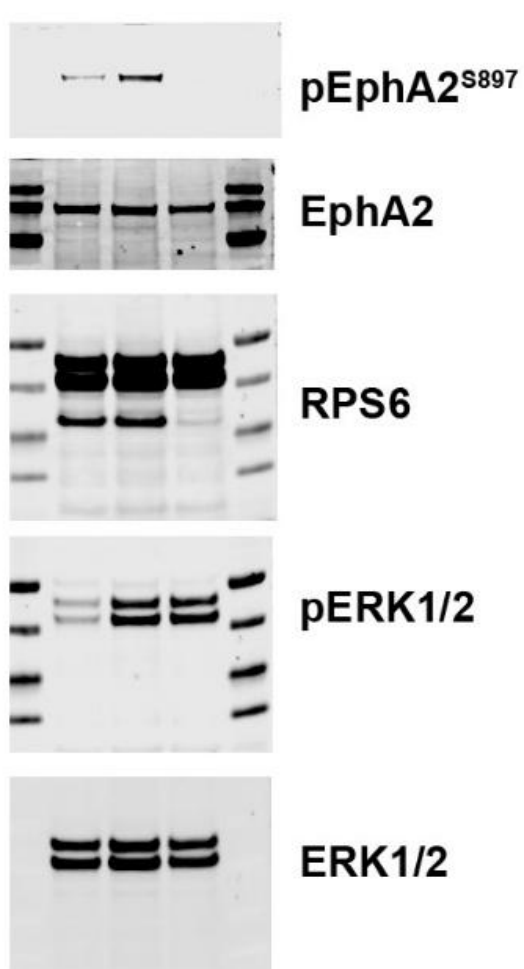

FIGURE 3D

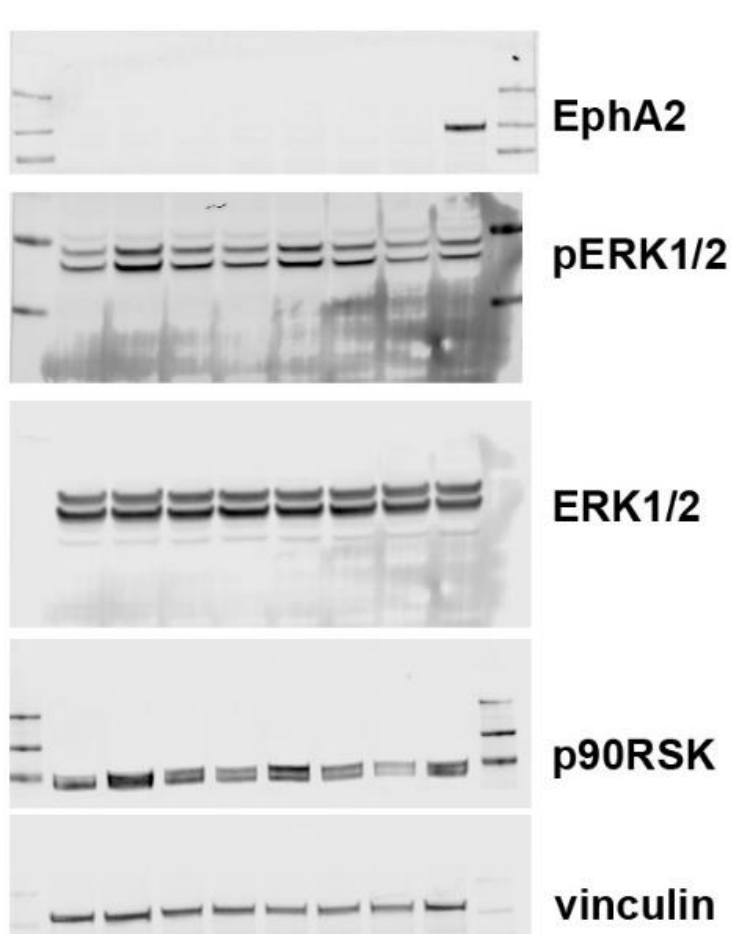

FIGURE 3L

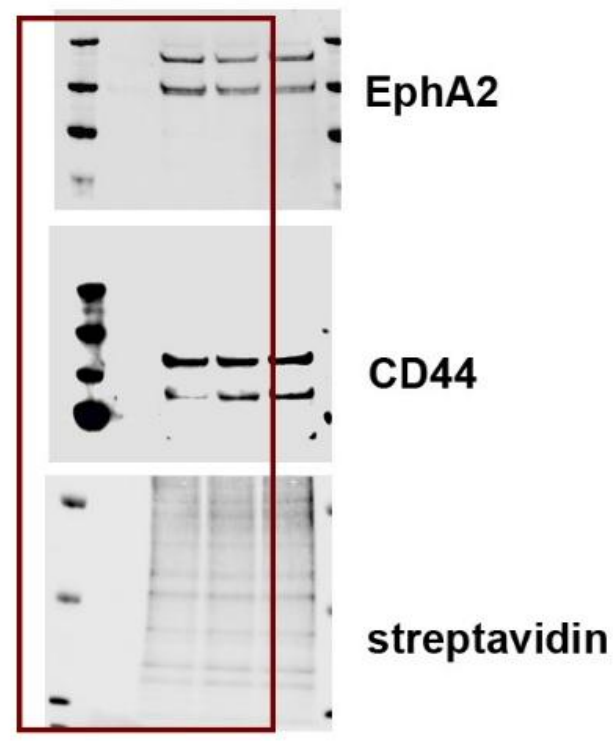

FIGURE 3N

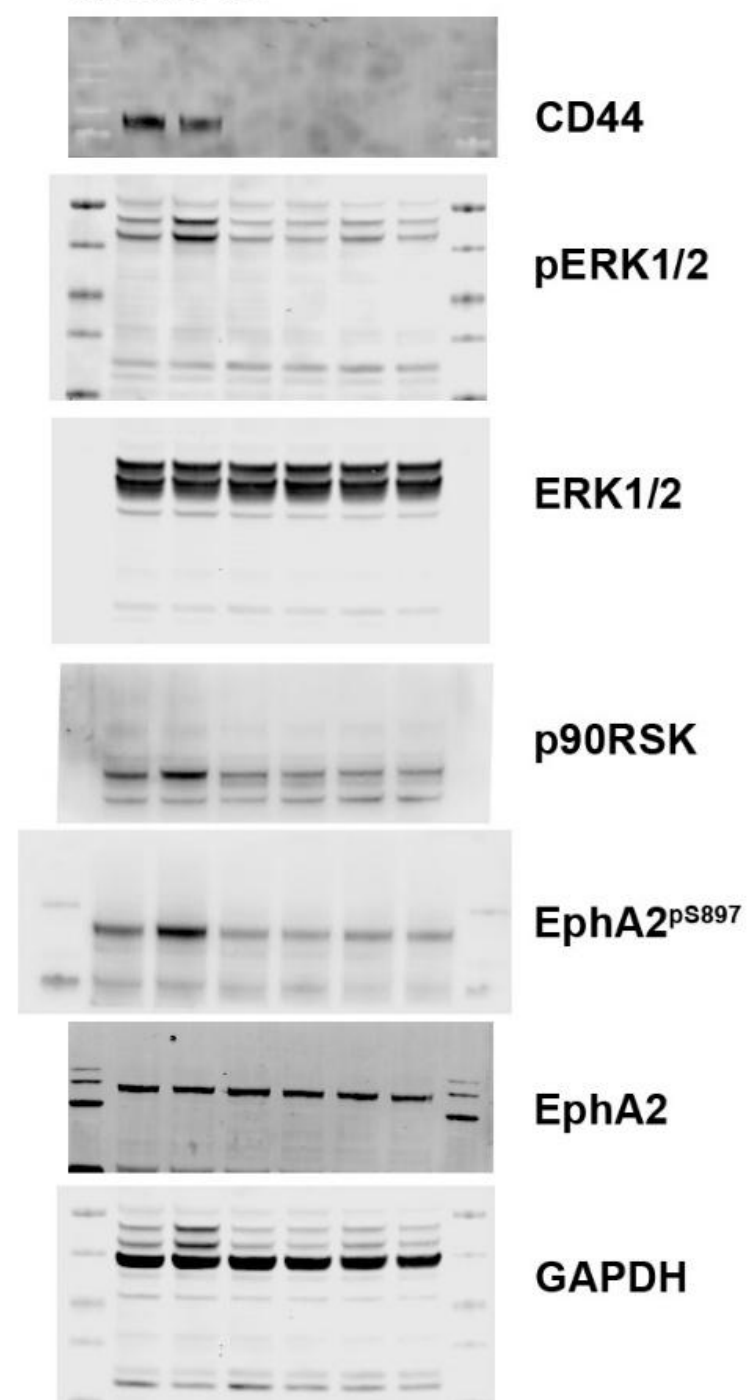

Supplement: Supplementary file 4 [file jcb_202507217_sourcedataf3.pdf]
